# Characterization of two *Pantoea* strains isolated from extra-virgin olive oil

**DOI:** 10.1186/s13568-018-0642-z

**Published:** 2018-07-10

**Authors:** Graziano Pizzolante, Miriana Durante, Daniela Rizzo, Marco Di Salvo, Salvatore Maurizio Tredici, Maria Tufariello, Angelo De Paolis, Adelfia Talà, Giovanni Mita, Pietro Alifano, Giuseppe Egidio De Benedetto

**Affiliations:** 1 Department of Biological and Environmental Sciences and Technologies (DiSTeBA), University of Salento, Via Provinciale Monteroni 165, 73100 Lecce, Italy; 2 Istituto di Scienze Delle Produzioni Alimentari-CNR, Via Provinciale Monteroni 165, 73100 Lecce, Italy; 3 Laboratory of Analytical and Isotopic Mass Spectrometry, Department of Cultural Heritage, University of Salento, Lecce, Italy

**Keywords:** *Pantoea septica*, *Stenotrophomonas rhizophila*, *Sporobolomyces roseus*, Olive oil microbiology, Fatty acid metabolism, Carotenoids, Bioemulsifier

## Abstract

The olive oil is an unfavorable substrate for microbial survival and growth. Only few microorganisms use olive oil fatty acids as carbon and energy sources, and survive in the presence of olive oil anti-microbial components. In this study, we have evaluated the occurrence of microorganisms in 1-year-stored extra-virgin olive oil samples. We detected the presence of bacterial and yeast species with a recurrence of the bacterium *Stenotrophomonas rhizophila* and yeast *Sporobolomyces roseus*. We then assayed the ability of all isolates to grow in a mineral medium supplemented with a commercial extra-virgin olive oil as a sole carbon and energy source, and analyzed the utilization of olive oil fatty acids during their growth. We finally focused on two bacterial isolates belonging to the species *Pantoea septica*. Both these isolates produce carotenoids, and one of them synthesizes bioemulsifiers enabling the bacteria to better survive/growth in this unfavorable substrate. Analyses point to a mixture of glycolipids with glucose, galactose and xylose as carbohydrate moieties whereas the lipid domain was constituted by C6–C10 β-hydroxy carboxylic acids.

**Electronic supplementary material:**

The online version of this article (10.1186/s13568-018-0642-z) contains supplementary material, which is available to authorized users.

## Introduction

The olive oil is an unfavorable substrate for microbial survival and growth (Ciafardini and Zullo 2002a, b; Brenes et al. 2007). Olive oils, in particular virgin and extra-virgin oils, contain small amounts of water in the form of mini-drops, and high content of various single and complex phenolic and glutaraldehyde-like compounds which are released during the malaxation phase of the olive oil extraction process, and possess strong antimicrobial activity (Brenes et al. 2007; Juven and Henis 1970; Fleming et al. 1973; Gourama et al. 1989). In this substrate microorganisms are often below the limit of detection with standard culture methods, and only few microorganisms are able to overcome the strong selective pressure of the olive oil antimicrobial compounds (Brenes et al. 2007; Ciafardini et al. 2017, Ciafardini and Zullo 2018; Medina et al. 2009), and to use olive oil fatty acids as sole carbon and energy sources. For this reason, information on olive oil microbiology has been limited for long time with very few studies published only during the last decade.

The content of water mini-drops in freshly produced virgin and extra-virgin olive oils may be different depending on method of extraction process of the olive oil and its storage for sedimentation (Koidis et al. 2008). After a few weeks or months, the mini-drops accumulate and can trap microorganisms whose survival depends on both the access to macro- and micro-nutrients and their susceptibility to the olive oil anti-microbial components (Koidis et al. 2008). In general, sporadic occurrence of microorganisms in olive oils can be the result of environmental contamination during their manufacture and storage, and, as such, considered unspecific. However, some studies have demonstrated the presence of a specific microflora mainly composed of yeasts belonging to the genera *Saccharomyces*, *Candida* and *Williopsis* in the suspended fraction of freshly produced olive oil (Ciafardini and Zullo 2002a, b, 2018), and occasionally moulds of the genus *Aspergillus*. On the other hand, there is limited information about the occurrence of bacteria (Koidis et al. 2008). Yeasts have contrasting roles in olive oil: on one hand they are thought to contribute to improvement of the organoleptic traits of the olive oil, on the other hand they can decrease its safety, integrity and taste (Ciafardini and Zullo 2018). Some dimorphic species can also be found among the unwanted yeasts in the olive oil. Some of these species are considered opportunistic pathogens of humans (Koidis et al. 2008; Zullo and Ciafardini 2008; Zullo et al. 2010).

Interestingly, olive oil microorganisms have a biotechnological potential. Indeed, fat/oil/grease-tolerant and/or metabolizing microorganisms are used for bioremediation of oily wastewaters and contaminated soils (Ammar et al. 2005; Azhdarpoor et al. 2014; Erguderet et al. 2000; Ettayebi et al. 2003; Kissi et al. 2001), and are valuable sources for enzymes, e.g., lipases, for industrial bioconversion of lipids, fats and oils into high-value products (Borrelli and Trono 2015; Sabirova et al. 2011). Fat/oil/grease-tolerant and/or metabolizing microorganisms may be also the source of biosurfactants, valuable molecules with pronounced surface and emulsification activities (Maier 2003; Singh et al. 2007; Van Hamme et al. 2006). Biosurfactants are a large group of various substances that can be categorized by their microbial origin and chemical composition into (i) glycolipids, (ii) fatty acids, phospholipids and neutral lipids, (iii) polymeric biosurfactants, (iv) particulate biosurfactants (Santos et al. 2016). These heterogeneous “green” compounds find applications in many fields of pharmaceutical industry, food industry, cosmetics, petroleum industry, environmental protection and agriculture (Santos et al. 2016; Singh et al. 2007).

In the present study we started with the identification and characterization of microorganisms in 1-year stored extra virgin olive oils, in order to investigate their adaptive strategies to grow/survive in this unfavorable environment also in view of their possible biotechnological exploitation. Then, focused our attention on two bacterial isolates, strictly related to the species *Pantoea septica* (Brady et al. 2010). Both these isolates produce carotenoids, and one of them synthesizes a mixture of bioemulsifiers: GC–MS and LC–MS analyses suggest they are glycolipids.

## Materials and methods

### Olive oils and microbiological media

Ten extra-virgin olive oils produced by oil mills located in Apulia region starting from blends of five different olive cultivars were used in this study (Table 1). They were stored in autoclaved dark bottles for 1 year at room temperature. After this period, the bottles were opened under sterile conditions, and checked for the presence of microorganisms.

**Table 1:** Properties of olive oil samples and microbial isolates

Extra-virgin olive oil sample	Geographical provenience	Olive cultivar	Microorganism found
#1	Laterza, Taranto	Leccino, Coratina, Ogliarola and Frantoio	*Sporobolomyces roseus*
#2	Mottola, Taranto	Leccino, Coratina, Ogliarola and Frantoio	*Stenotrophomonas rhizophila* *Pseudomonas cedrina*
#3	Martano, Lecce	Cellina di Nardò and Ogliarola	None
#4	Calimera, Lecce	Cellina di Nardò and Ogliarola	None
#5	Corigliano d’Otranto, Lecce	Cellina di Nardò and Ogliarola	None
#6	Galatone, Lecce	Cellina di Nardò and Ogliarola	None
#7	Lecce	Cellina di Nardò and Ogliarola	None
#8	Galatone, Lecce	Cellina di Nardò and Ogliarola	None
#9	Brindisi	Cellina di Nardò and Ogliarola	*Stenotrophomonas rhizophila* *Pseudomonas stutzeri*
#10	Casarano, Lecce	Cellina di Nardò	*Sporobolomyces roseus* *Pantoea septica*

Lysogeny Broth (LB) (10.0 g/L NaCl, 10.0 g/L tryptone, 5.0 g/L yeast extract, 15.0 g/L agar), and yeast extract peptone dextrose (YEPD) (10.0 g/L yeast extract, 20.0 g/L peptone, 20.0 g/L ^1^ d-glucose, 20.0 g/L agar) agar media were used for isolation of microorganisms from olive oil samples. The chemically defined M9 medium (6.78 g/L Na_2_HPO_4_, 3.0 g/L KH_2_PO_4_, 0.5 g/L NaCl, 1.0 g/L NH_4_Cl, 0.49 g/L MgSO_4_·7H_2_O, 0.011 g/L CaCl_2_, 15.0 g/L agar when requested) was used as a base to formulate either M9-0 medium (without glucose) or M9-oil medium replacing glucose with DMSO-dissolved extra-virgin commercial olive oil (Alài^®^, Agricola Nuova Generazione Società Cooperativa, Martano, Lecce, Italy), for growth in the presence of olive oil as sole carbon and energy source, and free-fatty acids determination.

The fatty acid composition of the Alài^®^ olive oil (expressed as µg/mL) is the following: palmitic acid (186.67 ± 15.21), palmitoleic acid (25.67 ± 1.81), stearic acid (23.20 ± 1.92), oleic acid (946.23 ± 68.34), linoleic acid (102.54 ± 9.20), linolenic acid (12.71 ± 1.10). This product was sterilized by autoclaving before use to avoid growth of endogenous microorganisms.

### Isolation and identification of microorganisms

LB or YEPD agar plates were inoculated with different volumes of the olive oil samples (20–200 μL), and incubated at 28 °C for 24 (bacteria) and 72 h (yeasts) under aerobic conditions. At the end of the incubation period, all colonies were counted using a 10× magnification lens, and the bacterial or yeast densities were expressed as colony-forming units (CFU)/mL. Each single bacterial and yeast colony was inoculated into LB and YEPD broth, respectively, and incubated at 28 °C until to middle logarithmic phase under rotary shaking at 180 rpm. All manipulations were carried out under sterile conditions in a laminar flow cabinet.

Conventional methods were used for phenotypic identification of microorganisms. Bacterial colonies were characterized considering a number of phenotypic traits: colony morphology (size, shape and pigmentation); cell morphology (Gram staining, size and shape); catalase and oxidase tests. Further metabolic tests were assessed by using API 20E (BioMèrieux), API 20NE (BioMèrieux), API 50CH (BioMèrieux) and BBL Enterotube II BD. Yeast colonies were classified by colour, shape, margin of colony and metabolic activities by using API 20 C AUX test (BioMèrieux).

Molecular identification of bacteria and yeasts was performed by 16S/18S rRNA gene sequence analysis. To achieve this purpose, bacteria and yeasts were grown under appropriate conditions in LB and in YEPD liquid media, respectively, to late logarithmic phase. After centrifugation at 3000 rpm for 20 min, pellets were re-suspended in 500 µL of SET buffer (75 mM NaCl, 25 mM EDTA, 20 mM Tris–HCl pH 7.5). Lysozyme was used at a final concentration of 1 mg/mL (w/v) and left to act at 37 °C for 1 h only for bacterial cells. Then sodium dodecyl sulphate (SDS) and proteinase K were added at a final concentration of 1% and 0.5 mg/mL respectively and incubated at 55 °C for 2 h in a water bath and periodically stirred. Total nucleic acids were extracted by phenol:chloroform:isoamylic alcohol (25:24:1 [v/v/v]) method according to standard procedures (Sambrook and Russel 2001) and RNase A (final concentration 15 µg/mL) was used to remove contaminant RNA. After the extraction, high-molecular weight DNA was used as template in PCR reactions to amplify the partial length 16S rRNA gene or the NS1/NS8 region of the 18S rRNA gene, respectively. The 16S rRNA encoding-genes were amplified by using a specific primers pair 16SE20-42-F (5′-TGGCTCAGATTGAACGCTGGCGG-3′) and 16SEB1488-R (5′-TACCTTGTTACGACTTCACC-3′), which were designed on the *Escherichia coli* 16S rRNA gene (Vigliotta et al. 2007). These primers target a 1400 bp-long DNA fragment. The 18S rRNA encoding-genes were amplified by using a couple of primers NS1-F (5′-GTAGTCATATGCTTGTCTC-3′) and NS8-R (5′-TCCGCAGGTTCACCTACGGA-3′) drawn on the *Saccharomyces cerevisiae* 18S rRNA gene (White et al. 1990). The resultant PCR product was long about 1700 bp. All PCR reactions were performed using a Biorad C1000 Touch Thermal Cycler. PCR products were separated by agarose gel in 1× TAE buffer (40 mM Tris–acetate, 1 mM EDTA, pH 8.0), recovered by using the Qiaex II Gel extraction kit (Qiagen) and sequenced by using the same primers pair utilized for the respective amplifications by MWG Biotech Customer Sequencing Service (Germany). The sequences of all bacterial and yeast isolates were compared with those of their closely related reference strains present in EzTaxon-e server (Kim et al. 2012) and in nucleotide BLAST database, respectively. Multiple sequence alignments between each pair of sequences were performed with ClustalW program at the Kyoto University Bioinformatic Center (http://www.genome.jp/tools/clustalw/) as previously described (Talà et al. 2013). Phylogenetic trees were constructed using the SeaView software (Gouy et al. 2010) according to the neighbour-joining (NJ) (Saitou and Nei 1987), maximum-parsimony (MP) (Sober 1983), and maximum-likelihood (ML) (Felsenstein 1981) methods and Kimura’s two-parameter algorithm (Kimura 1980). Tree robustness was determined by bootstrap analysis based on 1000 resamplings of data (Brown 1994).

The 16S/18S rDNA nucleotide sequences of all isolates were deposited at GenBank with the following accession numbers: *Pantoea septica* OOYS-10 (KJ534278), *Pantoea septica* OOWS-10 (KJ534279), *Pseudomonas stutzeri* OOYW-9 (KJ534280), *Pseudomonas cedrina* OOBS-2 (KJ534281), *Stenotrophomonas rhizophila* OOOWS-2 (KJ534282), *Stenotrophomonas rhizophila* OOOWS-9 (KJ534283), *Sporobolomyces roseus* OOPS-1 (KJ534284), *Sporobolomyces roseus* OOPS-10 (KJ534285). *P. septica* OOWS-10 was deposited in publicly accessible culture collection (WDCM945) of microorganisms of agricultural, industrial and environmental interest (COLMIA) at the Research Centre for Plant Protection and Certification, Council for Agricultural Research and Economics (CREA) with the strain number CREA-PAV 1867.

### BOX-PCR genomic fingerprinting

Bacterial DNA from the two isolates of *P. septica* and reference *E. coli* K12 strain FB8 was extracted as described above, and BOX-PCR genomic fingerprinting was carried out as previously described (Pizzolante et al. 2017; Versalovic et al. 1994) using the BOXA1-R primer (5′-CTACGGCAAGGCGACGCTGACG-3′). Amplification was performed with a BIO-RAD Thermal Cycler C1000 Touch using an initial denaturation step at 95 °C for 6 min, and subsequent 35 cycles of denaturation at 94 °C for 1 min, annealing at 53 °C for 1 min and extension at 65 °C for 8 min followed by a final extension at 65 °C for 16 min. PCR products were separated on a 1% (w/v) agarose gel in 1× TBE buffer (Sambrook and Russel 2001).

### Growth of microorganisms with olive oil as sole carbon and energy source

To test the ability of the isolated microorganisms to grow in the presence of olive oil as sole carbon and energy source, all strains were grown to middle logarithmic phase in M9 medium with glucose at 28° with rotary shaking under aerobic conditions. After incubation time, cells were centrifuged, washed twice, re-suspended in M9-0 medium (without glucose) and plated at appropriate dilutions on solid M9 medium supplemented with an olive oil:DMSO emulsion (1:9 v/v) to reach a final concentration of 1% (v/v) (M9-OO medium, where OO is the abbreviation for olive oil). Then, the microorganisms that exhibited growth on solid M9-OO medium, were inoculated into liquid M9-OO medium until middle logarithmic phase at 28 °C with rotary shaking. No growth of bacteria was observed either on solid M9-0 medium or in liquid M9-0 medium (without glucose or olive oil), which were used as negative controls.

### Free fatty acids extraction and determination from culture medium

Microorganisms were grown in liquid M9-OO medium to late logarithmic phase at 28 °C with rotary shaking under aerobic conditions. Before analyzing free fatty acid content at different time intervals (24, 30, and 48 h), the growth medium was acidified to pH 2.0 with 50% H_2_SO_4_ (v/v) as described (Yu et al. 2008). The resulting solution was extracted twice with an equal volume of ethyl acetate. The mixture was centrifuged at 5000×*g* for 15 min. The solvent was evaporated to dryness at 40 °C, under reduced pressure by a Rotavapor (RE120, Büchi Labortechnik AG, Postfach, Switzerland). After the obtained fatty acids were methylated with BF_3_/MeOH (12% v/v) at 100 °C for 10 min, the reaction was stopped by adding saturated NaHCO_3_ and extracted again with n-hexane as described (Lee et al. 2003a, b). Fatty acid methyl esters (FAMEs) were identified and quantified by gas chromatography–mass spectrometry (GC–MS) as described below.

### GC–MS analysis

FAMEs were analyzed using a GC–MS system (Shimadzu GC-17A ver. 3.0) with MS QP5050A, equipped with a DB-5 capillary column having 30 m length, 0.25 mm ID and 0.25 µm thickness. The operation conditions were similar to those previously described (Talà et al. 2013): the column temperature was 80 °C at the injection then programmed at 10 °C/min to 150 °C, at 5 °C min to 250 °C and maintained at 250 °C for 15 min. Split injection was conducted with a split ratio of 50:1, the flow-rate was 1 mL/min, carrier gas used was 99.999% pure helium, the injector temperature was 250 °C and the column inlet pressure was 74 kPa. The MS detection conditions were as follows: 250 °C interface temperature; ionization mode, EI+; electron energy, 70 eV; scanning method of acquisition, ranging from 30 to 450, for mass/charge (*m/z*) optimization. Spectrum data were collected at 0.5 s intervals. Solvent cut time was set at 2 and 45 min retention time enough for all fatty acids separation all. Compounds were identified by using online NIST-library spectra and published MS data. Moreover, FAME mix (C_8_–C_24_) authentic standard was used to confirm MS data. For quantitative assessment of the different free fatty acids, calibration curves using external standards were prepared using different concentrations of each FAME.

### Determination of fatty acid profile of *Pantoea* sp. strains

Exponentially growing bacteria were collected by centrifugation (8000×*g* at 4 °C for 15 min). The cells were washed twice with 1% NaCl (w/v) and lyophilized overnight (Freezone 4.5 L Dry System, Labconco 33 Co. Thermo Scientific). Lipids were extracted using the modified method of Bligh and Dyer (1959). Lyophilized powder (100 mg) was mixed with a total of 114 mL solvent added in this sequence: chloroform, methanol, water to achieve a final chloroform/methanol/water ratio of 1:2:0.8 (by vol). Samples were shaken for 15 s after addition of each solvent, and incubated overnight at 4 °C. After centrifugation at 6500×*g* for 10 min, the supernatant was transferred into a separating funnel, and phase separation of the biomass-solvent mixtures was achieved by adding chloroform and water to obtain a final chloroform/methanol/water ratio of 2:2:1.8 (by vol.). After settling, the bottom phase was collected. A portion of the total lipid extract was *trans*-esterified according to Eguchi et al. (2001) at 80 °C for 1 h using a solution of methanol/hydrochloric acid/chloroform 10:1:1 (v/v/v). After the addition of 1 mL water, the mixture was extracted twice with 3 mL hexane/chloroform 4:1 (v/v) to obtain FAMEs, which were analyzed using GC–MS.

### Extraction and analysis of carotenoids and isoprenoid quinones from *Pantoea* sp. strains

Carotenoids and isoprenoid quinones were extracted from *Pantoea* sp. strains as described by Nelis and De Leenheer (1989) with some modifications. To about 50 mg of freeze-dried cells, 2 mL KOH (60% w/v), 2 mL methanol and 5 mL of ethanolic pyrogallol (6% w/v) were added under vigorous vortex mixing. After a digestion time of 45 min at 70 °C, the tubes were cooled and 15 mL of NaCl (1% w/w) were added. The mixture was extracted with 15 mL of hexane/ethyl acetate (9:1 v/v). The upper layer was evaporated and the dry residue was dissolved in 100 µL of ethyl acetate and analyzed using HPLC.

### HPLC analysis

Analyses were carried out by Agilent 1100 HPLC as described by Fraser et al. (2000) with slight modifications. Carotenoids were separated using a reverse-phase C30 column (5 μm, 250 × 4.6 mm) (YMC Inc., Wilmington, NC, USA) with mobile phases consisting of methanol (A), 0.2% ammonium acetate aqueous solution/methanol (20:80 v/v) (B), and methyl tertiary butyl ether (C). The elution was as follows: 0 min, 95% A and 5% B; 0–12 min, 80% A, 5% B, and 15% C; 12–42 min, 30% A, 5% B, and 65% C; 42–60 min, 30% A, 5% B, and 65% C; 60–62 min, 95% A, and 5% B. The column was re-equilibrated for 10 min between runs. The flow rate was 1 mL/min, and the column temperature was maintained at 25 °C. The injection volume was 10 μL. Absorbance was registered by diode array at wavelengths of 475 nm for carotenoids and 295 nm for quinones. Compounds were identified by comparing their retention times and UV–visible spectra to authentic standards.

### Emulsification activity and surface tension

*Pantoea septica* OOWS-10 and OOYS-10 isolates, and *E. coli* strain FB8 were grown to confluence on LB plates under aerobic conditions. Then bacteria were harvested and resuspended in 2 mL PBS. Samples were then centrifuged at 4000 rpm for 10 min, and the supernatants were assayed for emulsifying activity against diesel fuel. Two-milliliter aliquots of supernatants (or HPLC fractions) were mixed with 1.4 mL of diesel fuel, and vortexed at high speed for 2 min. The emulsion was observed after letting the tubes stand at room temperature for 60 min. Ten microliter of 1% (w/vol) Sudan Black solution was added to the diesel fuel to increase contrast as described (Smith et al. 2016).

Surface tension of the solutions was measured with an FTA 1000 series Goniometer (First Ten Angstroms, USA) using the pendant drop method. The instrument has the capability of measuring IFT values of 0–2000 mN/m with accuracy of 0.5% and resolution of 0.1%. The instrument was calibrated against water, and measurements were performed in triplicate at 25 ± 0.1 °C.

### Liquid chromatography–mass spectrometry (LC–MS)

Supernatants (prepared as described above for emulsifying activity determination) and HPLC fractions were analyzed by a Surveyor MS Pump on line with a LCQ DECA XP Plus (Thermo Finnigan) mass spectrometer equipped with an ESI source. Separations were performed on an analytical 2.1- by 100-mm Thermo Scientific Accucore C18 reverse-phase column (particle size, 2.6 µm) protected with a C18-Security Guard cartridge, 2.1 × 10 mm packed with the same stationary phase. The injection volume was 2 µL. The mobile phase components were 10 mM ammonium formate brought to pH 4.6 with formic acid (A) and acetonitrile (B); the isolates were eluted according to the following linear gradient of B: 0 min, 10%; 1 min, 10%; 20 min, 80%; 24 min, 80%; 25 min, 5%; 30 min, 5% at flow rate of 0.2 mL/min. The pooled bioemulsifier containing fraction was eluted with the following linear gradient of B: 0 min, 5%; 2 min, 5%; 35 min, 80%; 39 min, 80%; 40 min, 5%; 45 min, 5%. MS was operated in full scan positive mode in the mass range from *m/z* 350 to 1500. The crude water-soluble supernatants containing bioemulsifiers were fractionated using HPLC. 100 µL aliquots of *P. septica* OOWS-10 extract were injected on a Symmetry300 C4 column (150 × 2.1 mm, particle size 3.5 µm; Thermo Scientific) and eluted at a flow rate of 200 µL min-1 using water (Solvent A) and LCMS-grade acetonitrile (solvent B) applying the following gradient of B: 0 min, 5%; 2 min, 5%; 35 min, 80%; 39 min, 80%; 40 min, 5%; 45 min, 5%. Fractions (0.5 ml) were collected and monitored by emulsification activity. The fractions with higher values of emulsifying activity were characterized by GC–MS (after acid hydrolysis) and LC–MS.

### Acid hydrolysis of the bioemulsifier and GC–MS analysis

Microwave assisted acid hydrolysis of the bioemulsifier containing eluate was done as described previously (Faraco et al. 2016). In brief, fractions were transferred in Teflon vials and dried, then 1 mL of 2 M trifluoroacetic acid was added. The acidic hydrolysis of the samples was then performed using a microwave oven Milestone model ETHOS (Sorisole, Bergamo, Italy) with the following program: power 500 W, temperature 100 °C, duration 30 min. After centrifugation at 10,000 rpm for 5 min, supernatant was transferred in a 2 mL vial and dried. Derivatization was carried out as described (Fiehn et al. 2000). Carbonyl moieties were protected by methoximation, using 50 μL of a 20 mg/mL solution of methoxyamine hydrochloride in pyridine at 30 °C for 90 min. Afterward, derivatization was carried out with 50 μL of *N*-methyl-*N*-trimethylsilyltrifluoroacetamide (MSTFA) at 60 °C for 30 min. One-microliter aliquots of these solutions were injected in splitless mode into a GC/MS system consisting of an autosampler, a Scion 456 gas chromatograph, and a Scion TQ triple quadrupole mass spectrometer (all Bruker Daltonics, Freemont, USA). The chromatographic separation was performed on a chemically bonded fused silica capillary column Br 5-MS column (Bruker Daltonics), 0.25 mm internal diameter, 0.25 µm film thickness, 30 m length, connected to a 2 m long deactivated fused silica capillary pre-column. Injection temperature was 280 °C, the interface was set to 300 °C, and the ion source was adjusted to 230 °C. Oven conditions: initial temperature 70 °C, 2 min isothermal, 6 °C/min up to 310 °C, 10 min isothermal. Carrier gas: He, constant flow 1.0 mL/min. Electron impact spectra were recorded at 70 keV in selected ion monitoring mode to detect trimethylsilylated methoximate samples. The sugar standards used for identification were glucose, mannose, galactose, rhamnose, fucose, ribose, arabinose, and xylose (Sigma-Aldrich). β-Hydroxy acids were determined as trimethylsilylated derivatives using the same derivatization protocol: data were analyzed with MS Workstation (Bruker Daltonics) and AMDIS software. Identification of β-hydroxy acids was performed with the Wiley MS library search and comparison with spectral data.

## Results

### Isolation and identification of microorganisms from olive oil samples

LB and YEPD agar media were used to isolate microorganisms from ten 1-year-stored extra-virgin olive oil samples (Table 1). Microbial count and isolation were carried out under aerobic conditions. Of the ten analyzed samples, four (#1, #2, #9, #10 in Table 1) were positive by culture with a total microbial (yeasts and bacteria) count of about 5.0 × 10^3^ CFU/mL, on average. No direct correlation between olive cultivars used to make up the blends of olive oils and occurrence of microorganisms was found.

The isolates were preliminary grouped into six morphotypes on the basis of cultural features. These isolates were designated with the OO abbreviation (for olive oil, source of isolation), followed by the initial of colony type (YS, yellow and smooth; YW, yellow and wrinkled; WS, white and smooth; OWS, opaque white and smooth; BS, brownish and smooth; PS, pink and smooth) and a number representative of the analyzed sample. Biochemical tests and nucleotide sequence analysis of 16S/18S rRNA genes were performed in order to establish the identity of all microorganisms. A total of three bacterial and one yeast taxa were represented in the analyzed olive oil samples including two strains belonging to *Enterobacteriaceae* (OOWS-10, OOYS-10), two to *Xanthomonadaceae* (OOOWS-2, OOOWS-9), two to *Pseudomonadaceae* (OOBS-2, OOYW-9), two to the genus *Sporobolomyces* (OOPS-1, OOPS-10).

**Fig. 1:**
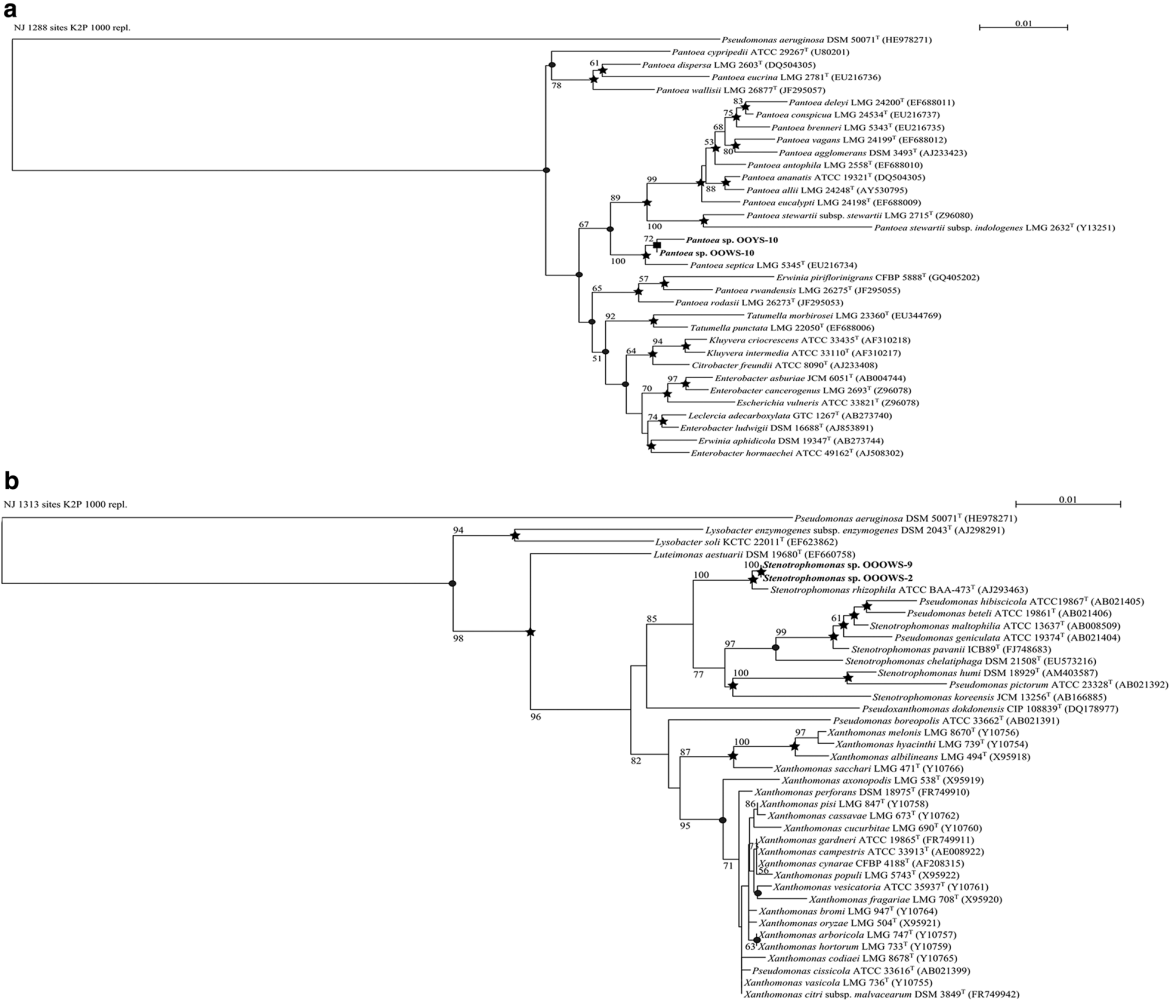
NJ phylogenetic tree based on 16S rRNA gene sequences showing the taxonomical positions of *Pantoea septica* strains OOWS-10 and OOYS-10 (**a**) and *Stenotrophomonas* spp. OOOW-2 and OOOW-9 (**b**) with respect to their closely related reference strains. Bootstrap values (expressed as percentages of 1000 replicates) of ≥ 50% are shown at branch points. Filled circle, filled square, star indicate that the corresponding nodes were also recovered in trees constructed using MP, ML, and both algorithms, respectively. *Pseudomonas aeruginosa* DSM 50071^T^ was used as an outgroup. Bar, 0.01 substitutions per nucleotide position

**Fig. 2:**
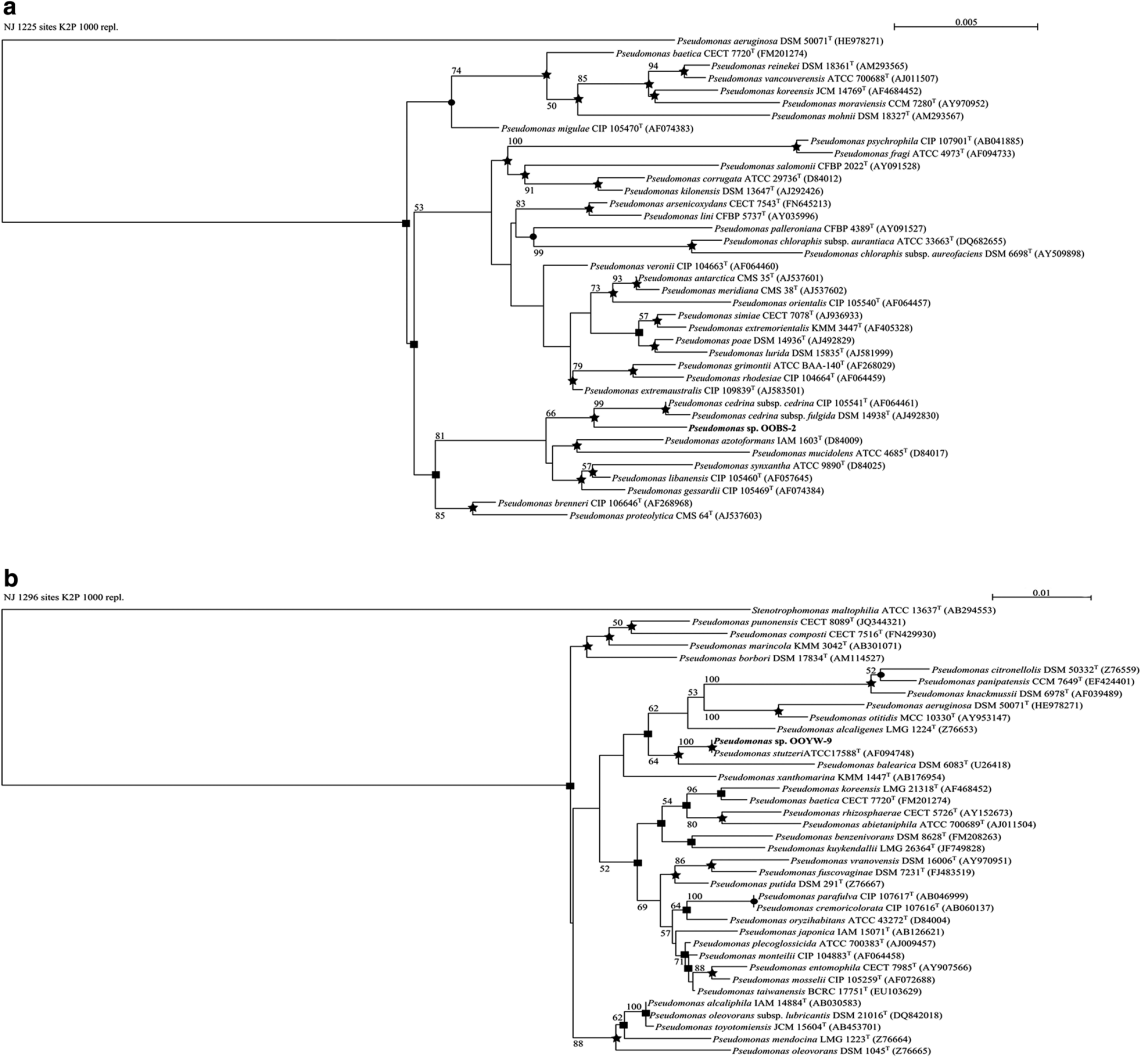
NJ phylogenetic tree based on 16S rRNA gene sequences showing the positions of *Pseudomonas* sp. OOBS-2 (**a**) and *Pseudomonas* sp. OOYW-9 (**b**) and some other related taxa. Bootstrap values (expressed as percentages of 1000 replicates) of ≥ 50% are shown at branch points. Filled circle, filled square, star indicate that the corresponding nodes were also recovered in trees constructed using MP, ML, and both algorithms, respectively. *Pseudomonas aeruginosa* DSM 50071^T^ and *Stenotrophomonas maltophilia* ATCC 13637^T^ were used as outgroups in **a** and **b** respectively. Bar, 0.005 and 0.01 substitutions per nucleotide position

**Fig. 3:**
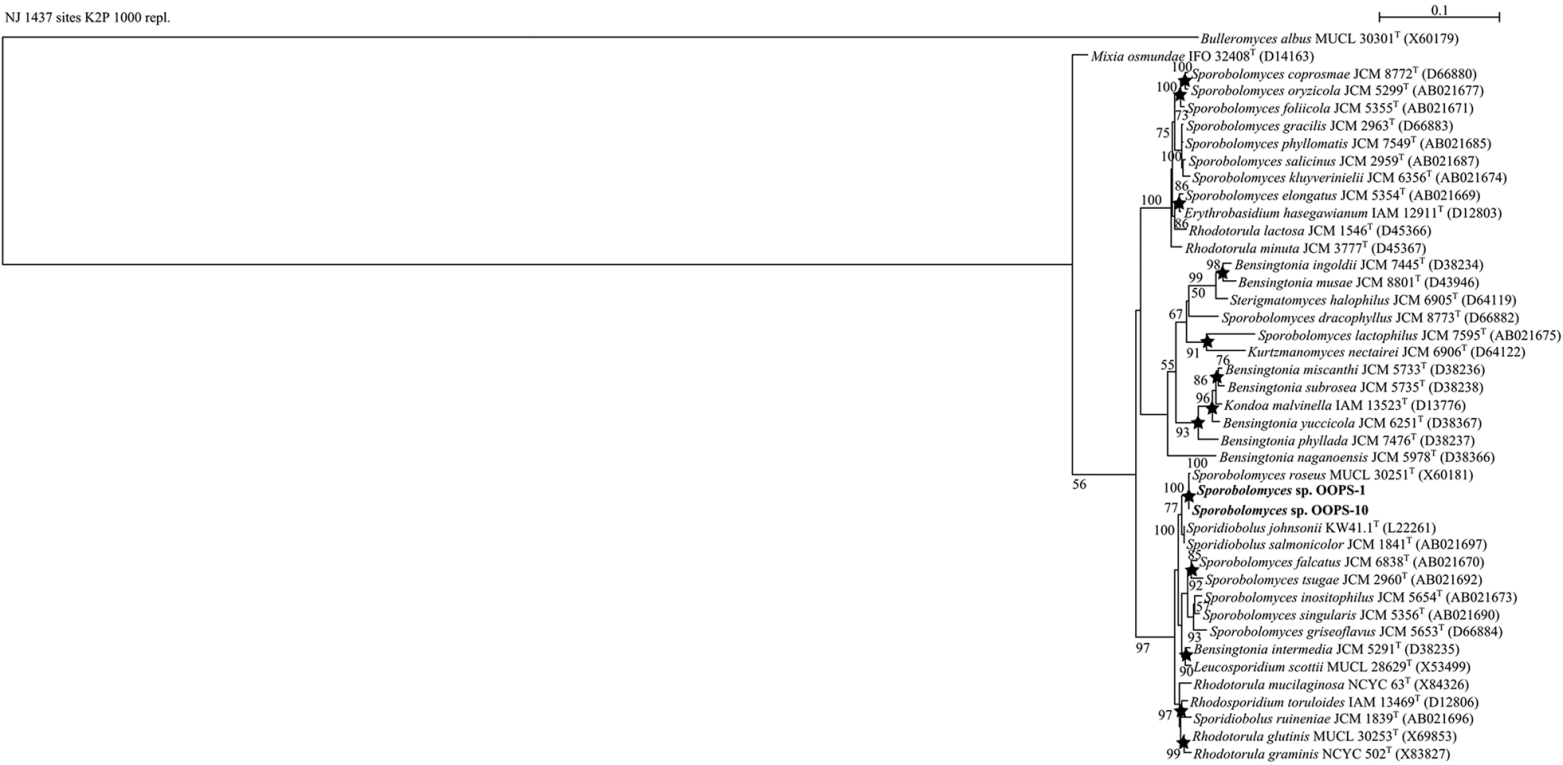
NJ phylogenetic tree based on 16S rRNA gene sequences showing the position of *Sporobolomyces roseus* OOPS-1, OOPS-10 and their strictly related reference strains. Bootstrap values (expressed as percentages of 1000 replicates) of ≥ 50% are shown at branch points. Filled circle, filled square, indicate that the corresponding nodes were also recovered in trees constructed using MP, ML, and both algorithms, respectively. *Bulleromyces albus* MUCL 30301^T^ was used as an outgroup. Bar, 0.1 substitutions per nucleotide position

Phylogenetic relationships between the 16S rRNA gene sequences of the olive oil bacteria and those of their strictly related reference strains are shown (Figs. 1, 2). The phylogenetic analysis of the *Enterobacteriaceae* tree (Fig. 1a) showed high similarity between OOWS-10 and OOYS-10 16S rRNA gene sequences and that of *Pantoea septica* (Brady et al. 2010). Biochemical tests confirmed the taxonomic assignment of the two isolates to this species (Additional file 1: Table S1). The phylogenetic data collocated OOOWS-2 and OOOWS-9 in the *Stenotrophomonas rhizophila* cluster (Fig. 1b). The two olive oil isolates shared the same phenotypic traits (Additional file 1: Table S2). The identification of *Stenotrophomonas rhizophila* in two unrelated olive oil samples suggests that its presence was not merely accidental in this substrate. The isolate OOBS-2 was positioned in the *Pseudomonas cedrina* phylogenetic branch (Fig. 2a). However, the biochemical markers failed to assign OOBS-2 unambiguously to *P. cedrina*. In fact, this microorganism shared some phenotypic traits with both *Pseudomonas cedrina* subsp. Cedrina (Behrendt et al. 2009; Dabboussi et al. 1999) and *Pseudomonas gessardii* (Verhille et al. 1999) (Additional file 1: Table S3). In contrast, 16S rRNA sequence analysis and biochemical data unambiguously assigned OOYW-9 to the species *Pseudomonas stutzeri* (Fig. 2b and Additional file 1: Table S4) (Lehmann and Neumann 1896; Sijderius 1946). Lastly, the 18S rRNA phylogenetic data collocated the yeast isolates OOPS-1 and OOPS-10 in the cluster of *Sporobolomyces roseus* (Fig. 3) (Bai et al. 2002; Nakase 2000). Biochemical traits were consistent with this assignment (Additional file 1: Table S5).

We then assayed their ability to grow in a mineral medium supplemented with a commercial extra-virgin olive oil (M9-OO) as a sole carbon and energy source. The fatty acid composition of the commercial extra-virgin olive oil used for these experiments is reported in “Materials and methods” section. All microbial isolates grew well either on solid or in liquid M9-OO media with growth rates during early exponential phase (*μ*) ranging from 0.10 to 0.40 (Additional file 1: Table S6). In this medium, *P. septica* OOYS-10 and OOWS-10, and *S. roseus* OOPS-1 and OOPS-10 isolates exhibited the highest growth rates. These results demonstrate that all microbial isolates were able to utilize olive oil fatty acids, although we cannot excluded the possibility that other olive oil components including oleuropein can also be used as some strains were positive to β-galactosidase activity (Additional file 1: Tables S2–S4).

### Characterization of the *P. septica* isolates OOWS-10 and OOYS-10

We next focused our attention on the two bacterial isolates, strictly related to the species *P. septica* because there is very limited information about this species and its physiology in the literature, and it has never been isolated before as a fat/oil/grease-tolerant and/or metabolizing microorganism.

We first decided to analyze the membrane fatty acid profile of the two isolates because it represents a useful chemo-taxonomical trait for classification of the *Pantoea* spp. (Mergaert et al. 1993) and, at the same time, it may be correlated with the utilization of olive oil fatty acids. Regarding the first point, it should be noted that information about the membrane fatty acid pattern of *P. septica* is currently missing. The GC–MS profiles of membrane-derived FAMEs were consistent with the taxonomical assignment of these two isolates to the genus *Pantoea* with a dominance of palmitic acid (C_16:0_) and an abundance of UFAs accounting for up to 40% of the total fatty acid content. The most represented UFAs in *P. septica* OOWS-10 and OOYS-10 were C_16:1ω7*c*_ (26.5 and 22.3%, respectively) and C_18:1ω7*c*_ (22.6 and 20.4%, respectively) (Additional file 1: Table S7).

The respiratory quinone profile was then analyzed by HPLC. The predominant isoprenoid quinone was ubiquinone-8 (Q-8) (~ 110 µg/g dw in *P. septica.* OOWS-10 and ~ 200 µg/g dw in *P. septica* OOYS-10); minor amount of ubiquinone-10 (Q-10) was also detected (~ 2 µg/g dw in OOWS-10 and ~ 6 µg/g dw in OOYS-10) (Additional file 1: Table S8). HPLC analysis of the pigments indicated that *P. septica* OOWS-10 and OOYS-10 produced lutein and a β-carotene pigment based on a comparison with authentic standards (Additional file 1: Table S8). The amount of lutein in *P. septica* OOWS-10 and OOYS-10 was 0.8 and 1.6 µg/g dw while β-carotene was 0.7 and 1.2 µg/g dw, respectively. It is interesting to note that yellow pigmentation of OOYS-10 was correlated with higher amount of carotenoids (Additional file 1: Table S8).

**Fig. 4:**
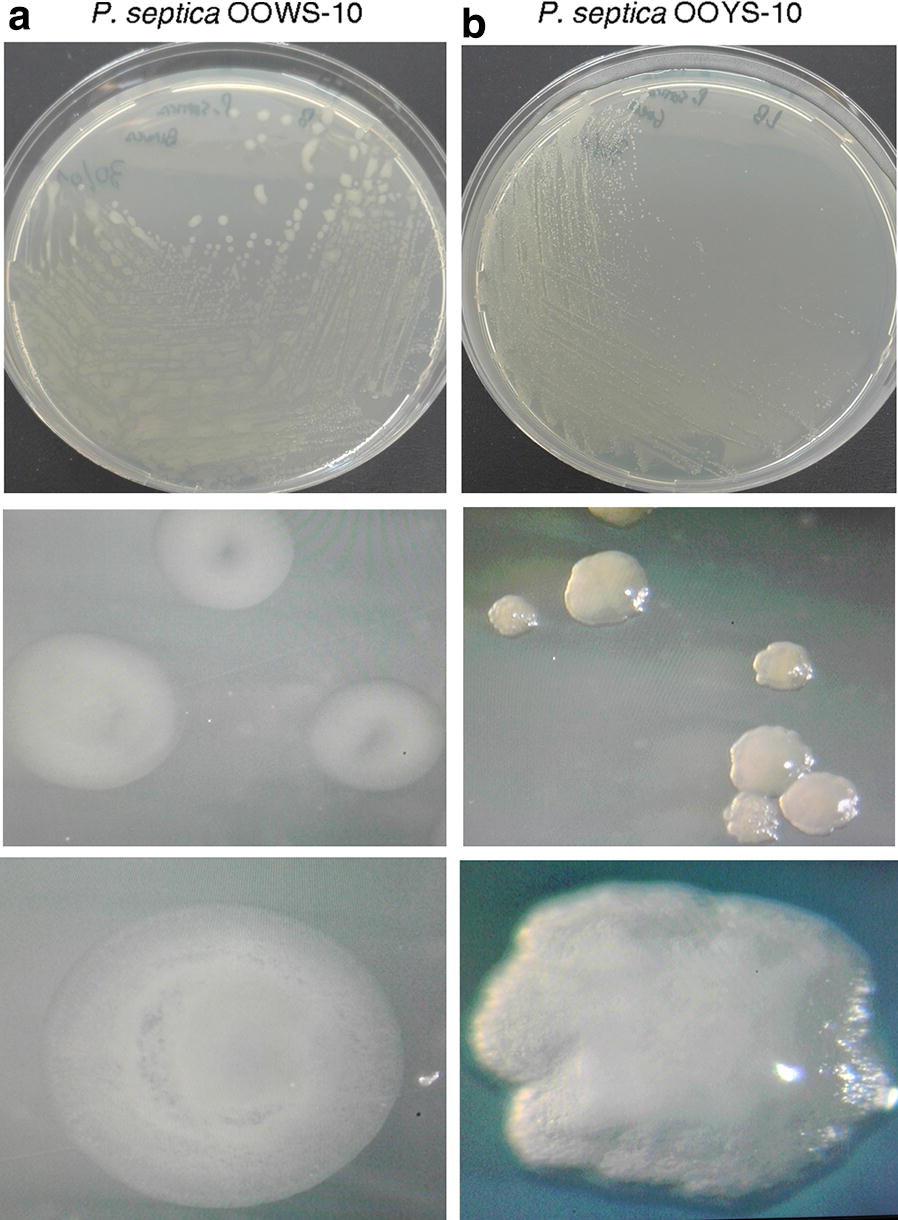
Colony phenotypes of *P. septica* isolates OOWS-10 (**a**) and OOYS-10 (**b**) on LB agar plates

On LB agar, the two isolates from olive oil formed different colonies for color, appearance, texture and opacity (Fig. 4). *P. septica* OOWS-10 showed large flat/crateriform, white, opaque, mucoid colonies with delineated margins (Fig. 4a); in contrast *P. septica* OOYS-10 formed small convex, yellow, translucent, dry leathery colonies with jagged edges (Fig. 4b). In spite of this remarkable difference in colony morphology, the two *P. septica* isolates were almost indistinguishable on the basis of biochemical tests (Additional file 1: Table S1) and 16S rRNA gene sequence analysis (Fig. 1a). To determine whether the two isolates are different morphotypes of the same *P. septica* strain or different strains belonging to the same species, we carried out BOX-PCR fingerprinting analysis. This technique is based on PCR amplification using a single primer that targets the repetitive BOX regions scattered in the genome of bacteria and results in strain-specific fingerprinting (Koeuth et al. 1995; Louws et al. 1994). It was successfully used to analyze the microdiversity of bacterial communities (Berg et al. 2005). The fingerprints of *P. septica* OOWS-10 and *P. septica* OOYS-10 were composed of 8–10 major bands with sizes ranging from about 500 to 3000 bp with some evident difference in genomic pattern between the two isolates (Additional file 1: Fig. S1). This result seems to suggest that the two isolates of *P. septica* are distinct strains.

### Olive oil fatty acids utilization by *P. septica*

**Fig. 5:**
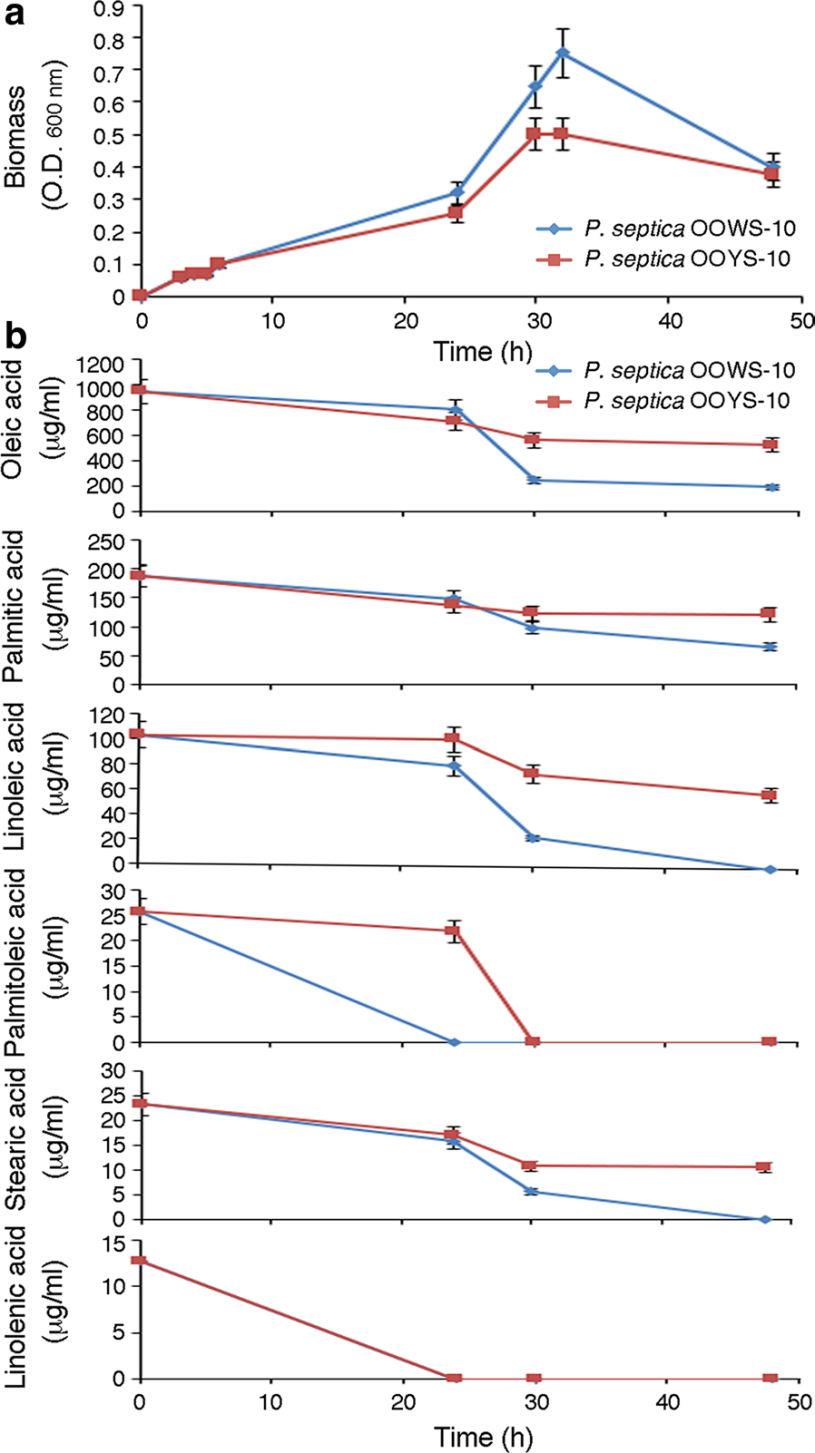
Growth curves of *P. septica* isolates OOWS-10 and OOYS-10 in M9-OO medium (**a**) and microbial utilization of Alài^®^ extra-virgin olive oil fatty acids as determined by GC–MS (**b**)

To gain more insight into the adaptive strategies of *P. septica* to grow/survive in an environment unfavorable for microbial growth, we analyzed the ability of the two isolates, OOWS-10 and OOYS-10, to utilize the olive oil fatty acids. To this purpose, the two isolates were individually cultivated in M9-OO medium (Fig. 5a), and the exhausted medium was harvested at different time intervals and analyzed by GC–MS (Fig. 5b). Both isolates were able to grow by utilizing the olive oil fatty acids albeit at a different extent. In particular, unsaturated fatty acids (UFAs) were rapidly utilized, and utilization of olive oil fatty acids was correlated with growth curves in M9-OO. For instance, with respect to *P. septica* OOYS-10, the isolate OOWS-10 reached higher final biomass values (Fig. 5a) and also exhibited higher extent of olive oil fatty acids utilization at later time points (Fig. 5b).

**Fig. 6:**
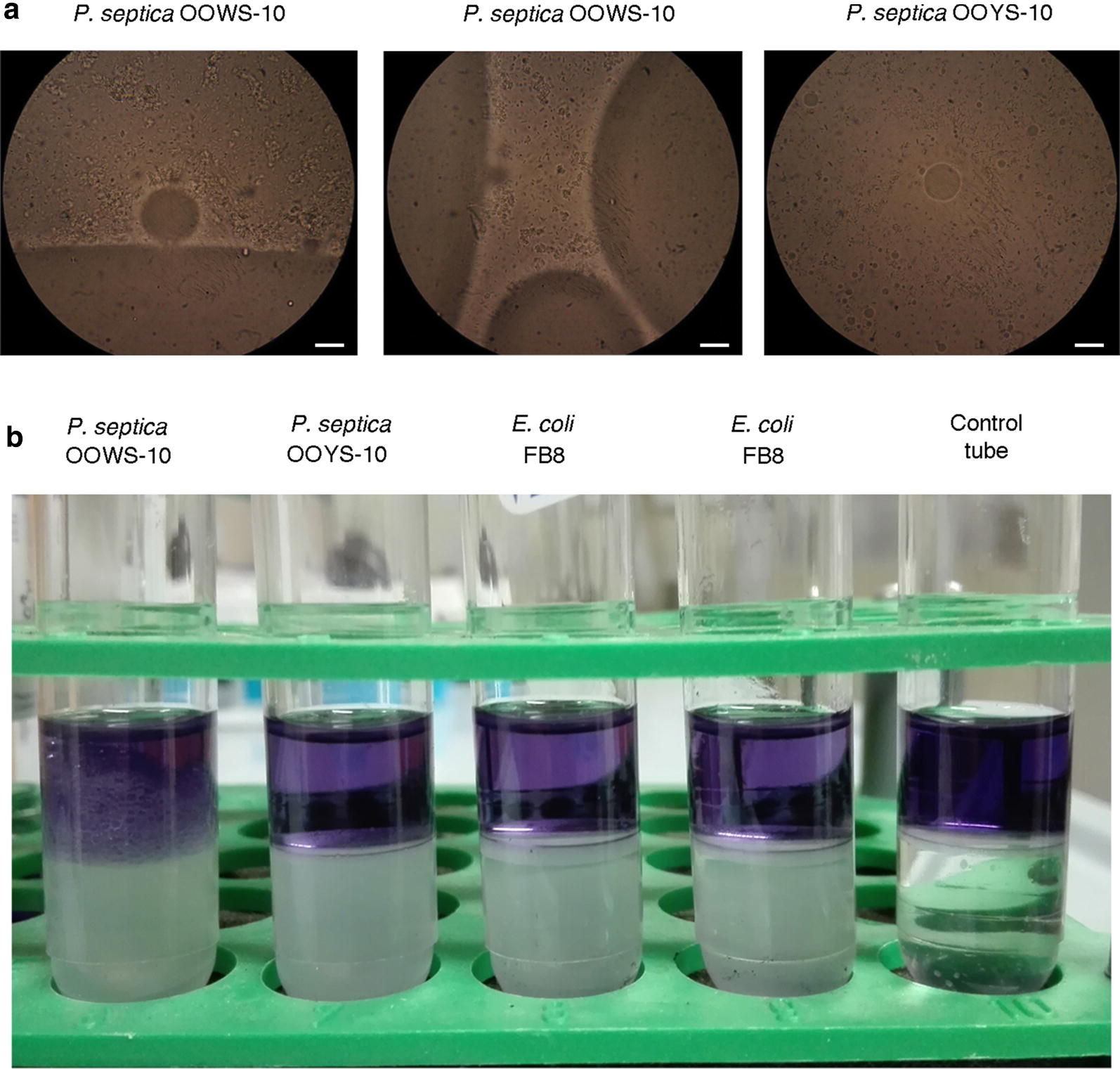
Emulsification activity associated with *P. septica* isolates OOWS-10. **a** Behaviors of *P. septica* OOWS-10 and OOYS-10 in an olive oil–water mixture. Note the tendency of *P. septica* OOWS-10 to form clusters at the interface of the oil–water mixture. The white bars represent 10 μm. **b** Emulsification activity assay. *P. septica* OOWS-10 and OOYS-10 were grown to confluence on LB agar, harvested and resuspended in PBS. Bacteria were removed by centrifugation and equal volume of bacteria supernatants and diesel fuel containing the lipophilic dye Sudan Black were then mixed, and allowed to separate in a test tube. *E. coli* strain FB8 was used as a negative control

The macro-morphology of *P. septica* OOWS-10 (Fig. 4), and its efficiency in metabolizing the olive oil fatty acids (Fig. 5) were suggestive of an ability to produce bioemulsifiers molecule(s), similarly to other fat/oil/grease-tolerant and/or metabolizing microorganisms (Santos et al. 2016). Indeed, preliminary observation by phase contrast microscopy indicated an aptitude of these bacteria to form clusters at the interface of an olive oil–water mixture (Fig. 6a, left and center). This property that could not be observed with *P. septica* OOYS-10 (Fig. 6a, right) was indicative of an ability of *P. septica* OOWS-10 to produce bioemulsifiers.

**Fig. 7:**
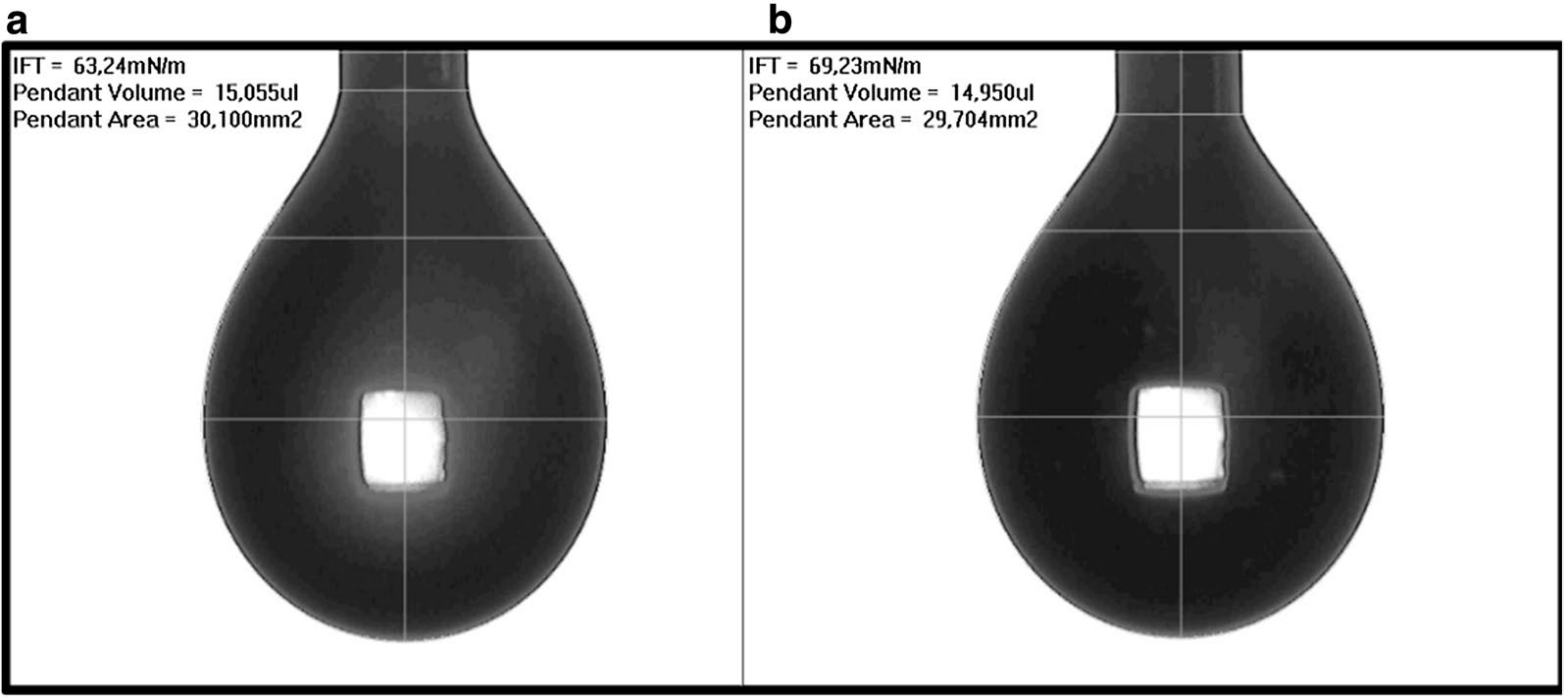
Surface tension measurements with pendant drop tensiometry: grayscale image, drop data and resulting surface tension of supernatants of *P. septica* OOWS-10 (**a**) and *P. septica* OOYS-10 (**b**)

To test this hypothesis, we verified the emulsifying activity of the two isolates against diesel fuel (Fig. 6b). The assay was performed with supernatants: bacteria were grown to confluence on LB agar, harvested and resuspended in PBS. Bacteria were removed by centrifugation and equal volume of bacteria supernatants and diesel fuel containing the lipophilic dye Sudan Black were then mixed, and allowed to separate in a test tube. *E. coli* strain FB8 was used as a negative control. In this assay, *P. septica* OOWS-10 demonstrated emulsifying properties. This behavior was not observed with the other bacterial strains. This qualitative assay was confirmed by surface tension measurements: as can be seen in Fig. 7 surface tension of *P. septica* OOWS-10 supernatant was about 63 mN/m, value lower than both the one recorded for *P. septica* OOYS-10 supernatant in the same conditions and the PBS control (Additional file 1: Fig. S2), suggesting the presence of poor bioemulsifiers.

### Characterization of bioemulsifiers from *P. septica*

**Fig. 8:**
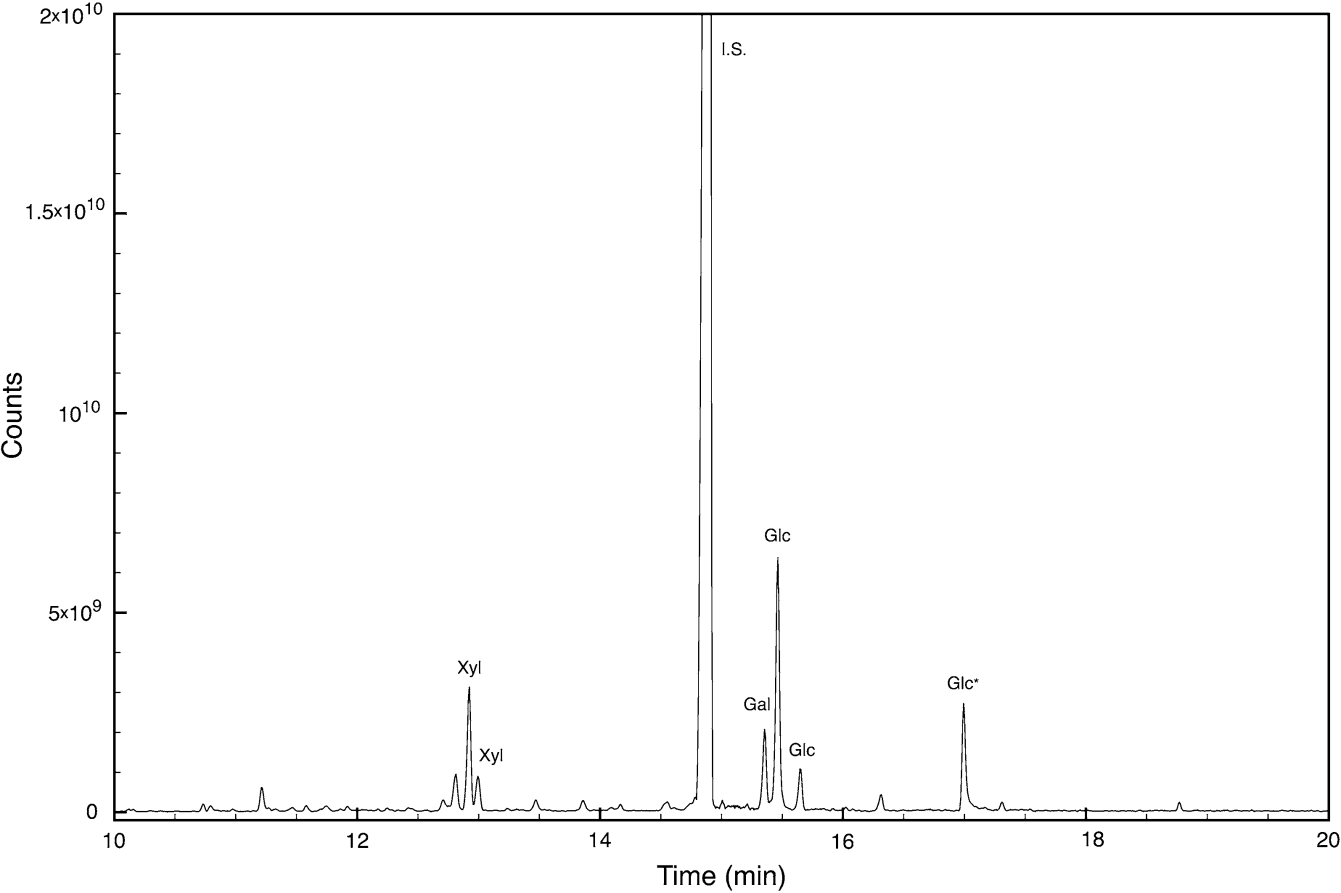
GCMS chromatogram showing the carbohydrate content of *P. septica* OOWS-10 supernatant fraction having emulsifying activity after microwave assisted acid hydrolysis

**Fig. 9:**
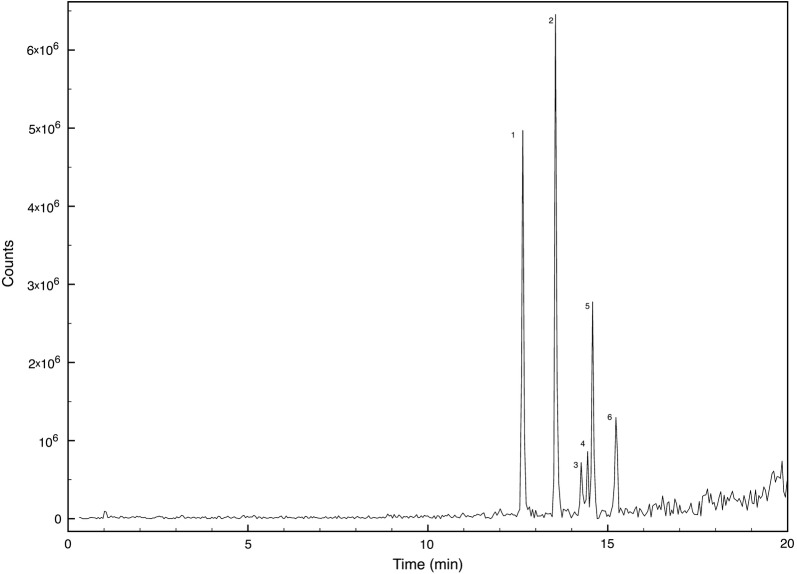
Total ion chromatogram of glycolipid homologues produced by *P. septica* OOWS-10. Peaks are numbered according elution order as reported in Table 2

Supernatants were also analysed by HPLC–ESI–MS in positive mode and the profiles confirmed the differences between the isolates (data not shown). However, it was not possible to identify in the chromatogram ions attributable to the known rhamnolipids reported for other *Pantoea* strains (Vasileva-Tonkova et al. 2007; Behrens et al. 2016; Rooney et al. 2009). As a result, *P. septica* OOWS-10 supernatant was fractionated by HPLC on a C4 column. Among the different fractions collected, only two showed emulsifying activity, the first from 15 to 17 min, and the second from 17 to 19 min. These two fractions were pooled and an aliquot was dried, and subjected to microwave assisted acid hydrolysis. The carbohydrate moieties in the produced bioemulsifiers were determined by GC–MS as ethoximate trimethylsilylated derivatives. Figure 8 shows the selected ion monitoring chromatogram: the monosaccharide composition was identified as xylose (46%), galactose (11%), and glucose (41%). As expected, sugars were detected only after acid hydrolysis. The hydroxy fatty acid composition was determined by GC–MS. Additional file 1: Fig. S3 shows the extracted ion chromatogram of *P. septica* OOWS-10 hydrolysate in the mass region *m/z* 230–350 where the β-hydroxy acids identified as di-trimethylsilylated compounds have been labeled. Another aliquot was analyzed by HPLC–MS. Only peaks absent from the other fractions and from *P. septica* OOYS-10 supernatant were considered and Fig. 9 shows the relevant extracted chromatogram. The molecular formula of each component was inferred from the pseudomolecular ion *m/z* and its fragmentation. As glucose and galactose could not be distinguished both moieties were labeled Hex. Peaks 1 and 2 at 12.64 and 13.54 min correspond to disaccharides, the former dixylose and the latter dihexose, having β-hydroxy fatty acids with a chain of 8 and 10 carbon atoms, respectively, whereas the other peaks are monosaccharides with different β-hydroxy fatty acids as lipid moieties. In particular the peak at 14.26 is a glycolipid with a hexose and two C6 units, both peaks 4 and 5 have a C6 and a C8 β-hydroxy fatty acids linked to a xylose and a hexose, respectively, and the last identified peak at 15.23 min is a monohexose bioemulsifier with a lipid backbone consisting of two C10 chains. In Table 2 the mass of the pseudomolecular ion, the suggested formula and relative percentages obtained using the peak area were listed and labeled according to the position in Fig. 9.

**Table 2:** Chemical structure and relative abundances of the glycolipids homologues produced by *P. septica* OOWS-10

Peak number	Glycolipid	Retention time (min)	[M + H]^+^ (m/z)	Peak area (counts/10E5)	Peak area (%)
1	Xyl–Xyl-C8	12.64	423.4	206	33.0
2	Hex–Hex-C10	13.54	511.5	246	39.4
3	Hex-C6-C6	14.26	407.5	23	3.7
4	Xyl-C8-C6	14.44	405.5	18	2.9
5	Hex-C8-C6	14.58	435.5	72	11.5
6	Hex-C10-C10	15.23	519.5	60	9.6

Xyl stands for a xylose molecule whereas Hex stands for a Glucose or a Galactose molecule; Cn stands for an alkane fatty acid molecule with n carbons in carbon chain length

## Discussion

In this study we have characterized eight microbial strains (six bacteria and two yeasts) from 1-year-stored extra-virgin olive oils, which were isolated under aerobic conditions. Two strains belonging to *Enterobacteriaceae* were assigned to the species *P. septica*. *Pantoea* is a genus of Gram-negative bacilli that have been shown to be either beneficial or harmful in association with plants (Walterson and Stavrinides 2015). There is very limited information about the species *P. septica*, although strains belonging to this species were implicated in nosocomial septicaemia outbreak in the USA in 1971 (Brady et al. 2010). Recently, the genome sequence of *P. septica* strain FF5 has been published (Lo et al. 2015). The occurrence of *P. septica* in olive oil demonstrates a high adaptability of this bacterium to different environments and substrates, and its biotechnological potential.

Two strains belonging to *Xantomonadaceae* were assigned to the species *Stenotrophomonas rhizophila* cluster. *S. rhizophila* is a plant growth promoting microorganism (PGPM) that lives in the rhizosphere of many plants, produces the plant growth hormone indoleacetic acid, and possesses antagonistic activity against plant-pathogenic fungi (Wolf et al. 2002; Suckstorff and Berg 2003; Schmidt et al. 2012). This species is resistant to cold and desiccation, and there is evidence that it is able to promote growth of a wide variety of crops in saline soils (Egamberdieva et al. 2011).

Among the two strains belonging to *Pseudomonadaceae*, one was related to *Pseudomonas cedrina* and *Pseudomonas gessardii*, but could not be assigned unambiguously to one or the other species, while the other was assigned to the species *Pseudomonas stutzeri*. This bacterium is widely distributed in natural environments and takes advantage of a great metabolic versatility (Lalucat et al. 2006). It is involved in environmentally important metabolic activities. Some of its major tasks are metal cycling and degradation of biogenic and xenobiotic compounds (Lalucat et al. 2006). Interestingly, literature also reports many crude oil-, oil derivative-, and/or aliphatic hydrocarbon-degrading *P. stutzeri* strains (Criddle et al. 1990; Janiyani et al. 1992; Pucci et al. 2000; Joo et al. 2001; Dijk et al. 2003; Hou et al. 2004).

The two yeast isolates were assigned to the species *Sporobolomyces roseus*. *S. roseus* is one of the most common phylloplane yeasts (Bai et al. 2002; Derx 1930; Nakase 2000). This pink yeast is extensively used for biotechnological purpose as a biocontrol agent, a lignin degrader, a protease and urease producer, a source of carotenoids for human and animal diet (Jahns 1995; Abranches et al. 1997; Filonow 2001; Kosikova and Slavikova 2004; Breierova et al. 2008). At the same time, it should be also pointed out this yeast has been also associated with disease in dogs and humans (Saey et al. 2011; McNicholas et al. 2012).

Although no direct correlation between olive cultivars (used to produce the olive oils) and occurrence of microorganisms was found in our analysis (Table 1), it is reasonable to assume that the origin of detected microorganisms may be the olive carposphere if we consider the general characteristics of the isolated microorganisms. For instance, *Pantoea* spp. were consistently found in the olive mesocarp of plants subjected to different cultural practices (Pascazio et al. 2015). Nevertheless, it is not possible to rule out the possibility that, at least in some cases, accidental microbial contamination may have occurred coming from staff and/or processing plant.

It should be also noted that although some bacteria could be isolated from the olive oil samples, their presence could be underestimated, as the isolation agar media were incubated for only 24 h. This period may be too short to “resuscitate” bacteria that may have been stressed/damaged in 1-year stored olive oils. In addition, the short incubation period may have favored the isolation of fast-growing species. Finally, it should be pointed out that both microbial count and isolation were performed under aerobic conditions. Indeed, to the best of our knowledge, the presence of strictly anaerobic microorganisms was not documented so far in other studies on stored olive oil, which, instead, mainly report the presence of aerobic or facultative anaerobic microorganisms (see “Introduction”). As the olive oil storage is expected to establish nearly anaerobic conditions, it would be interesting in the future to evaluate the eventual presence of strictly anaerobic bacteria in this substrate.

Notably, all microbial isolates were able to utilize the olive oils fatty acids as sole carbon and energy source for growth suggesting an adaptive strategy to grow/survive in this unfavorable substrate. Although the capability of using efficiently olive oil fatty acid for growth is expected for *S. roseus*, a yeast species that is also industrially used for production of UFAs (Cui et al. 2012), this capability is rather new for the other isolated microorganisms (i.e., *P. septica*, *S. rhizophila*, *P. stutzeri* and *Pseudomonas* sp. OOBS-2). However, very recently, a bacterial consortium that degrades cooking oil has been isolated in wastewater samples, by enrichment in olive cooking oil (Nzila et al. 2016). This consortium is formed by five bacterium species including *S. rhizophila*, *Sphingobacterium* sp. and three *Pseudomonas* species (*Pseudomonas libanensis*, *Pseudomonas poae* and *Pseudomonas aeruginosa*), can degrade the free fatty acids palmitic, stearic, oleic, linoleic and linolenic acids, and exhibit high levels of extracellular lipase activity. The occurrence of *S. rhizophila* and *Pseudomonas* species in our olive oil samples is thus consistent with this finding. Beside, it is worth noting the ability of *S. rhizophila* strain PM-1 to degrade crude oil and polycyclic aromatic hydrocarbons (Kumar and Manjunatha 2016; Virupakshappa et al. 2016) suggesting the utility of the isolated microorganisms in removal of exhausted cooking oil from wastewater, and in bioremediation of petroleum hydrocarbon contaminated environments. Indeed, as noticed by Margesin et al. (2003), the capability of microorganisms to degrade hydrocarbons and lipids is related to the fact that similar enzymes are involved in both degradation processes. In fact, the initial step of hydrocarbon oxidation produces primarily alcohols that are further converted to the corresponding fatty acids, which are metabolized through the β-oxidation pathway.

In this study we focused our attention on the two olive oil isolates belonging to the species *P. septica* for their ability to synthesize appreciable levels of carotenoids and poor bioemulsifiers enabling the bacteria to emulsify the olive oil and survive/growth in this unfavorable substrate. The production of carotenoids may be related to the metabolic adaptation of the microorganism to the olive oil environment. Indeed, it is well known that the utilization of long-chain fatty acids as main carbon source for growth stimulates H_2_O_2_ emission in aerobic bacteria and mitochondria, and that the respiratory complex III and the electron transfer flavoprotein (ETF) and ETF-oxidoreductase are likely sites of reactive oxygen species (ROS) production (Seifert et al. 2010). It is therefore conceivable that lutein and β-carotene are produced by the *P. septica* isolates to counteract the detrimental effects induced by ROS on cell physiology and metabolism, due to their ability to scavenge ROS (El-Agamey et al. 2004). In this regard, it is worth of noticing that carotenoid-defective mutant of *Pantoea* sp. YR343, a microorganism isolated from the rhizosphere of *Populus deltoids*, was defective in root colonization suggesting that carotenoids are important for plant association and/or rhizosphere survival (Bible et al. 2016). The evidence that the yeast *S. roseus*, which we also isolated from olive oil, is used for industrial production of carotenoids further support the link between long-chain fatty acid β-oxidation and carotenoid biosynthesis. Altogether these findings also indicate that the olive oil microorganisms such as *P. septica* could be proposed for industrial production of carotenoid compounds. Indeed, chemical synthesis of carotenoids is challenging and costly, while extraction from plants is also laborious and often limited by the availability of the sources. There exists a demand for microbial production of carotenoids by fermentation (Cheng 2007).

The presence of bioemulsifiers is the most notable trait of *P. septica* OOWS-10, distinguishing it from the other isolate, *P. septica* OOYS-10, and accounting for different phenotype of the two isolates on LB agar. A simple fractionation was carried out by reverse phase HPLC on a C4 column and only the eluate from 15 to 19 min contained bioemulsifiers. GC–MS analysis revealed a peculiar carbohydrate composition: no rhamnose could be identified in the hydrolysate as already observed with *Pantoea ananatis* BRT175 which produces glycolipid that incorporates a not identified hexose rather than rhamnose (Smith et al. 2016). In the present study a mixture of monosaccharides was detected as observed for glycolipids produced by bacteria *Halomonas* sp. grown in hexadecane (Pepi et al. 2005).

Whereas the carbohydrate and fatty acid composition of the bioemulsifier has been determined by GC–MS, the final formula assignment was made by HPLC–ESI–MS analyses, carried out solely on the eluate showing emulsifying activity. These analyses confirmed the presence of different glycolipids composed of β-hydroxy fatty acids with 6, 8, and 10 carbon atoms and pentoses or hexoses as hydrophilic head. In brief, these results suggested that the bioemulsifiers produced by *P. septica* OOWS-10 were glycolipids, in which disaccharides linked to a single β-hydroxy fatty acid chain were the main components. These bioemulsifiers could have potential application in bioremediation of olive oil waste.

## Additional file

**Additional file 1.** Additional tables and figures.

## Abbreviations

LC–MS: liquid chromatography–mass spectrometry
LB: Lysogeny Broth
YEPD: yeast extract peptone dextrose
CFU: colony-forming units
SDS: sodium dodecyl sulphate
FAMEs: fatty acid methyl esters
GC–MS: gas chromatography–mass spectrometry
MSTFA: *N*-methyl-*N*-trimethylsilyltrifluoroacetamide

## References

[CR1] Abranches J, Morais PB, Rosa CA, Mendonça-Hagler LC, Hagler AN (1997). The incidence of killer activity and extracellular proteases in tropical yeast communities. Can J Microbiol.

[CR2] Ammar E, Nasri M, Medhioub K (2005). Isolation of phenol degrading *Enterobacteria* from the wastewater of olive oil extraction process. World J Microbiol Biotechnol.

[CR3] Azhdarpoor A, Mortazavi B, Moussavi G (2014). Oily wastewaters treatment using *Pseudomonas* sp. isolated from the compost fertilizer. J Environ Health Sci Eng..

[CR4] Bai FY, Zhao JH, Takashima M, Jia JH, Boekhout T, Nakase T (2002). Reclassification of the *Sporobolomyces roseus* and *Sporidiobolus pararoseus* complexes, with the description of *Sporobolomyces phaffii* sp. nov. Int J Syst Evol Microbiol.

[CR5] Behrendt U, Schumann P, Meyer JM, Ulrich A (2009). *Pseudomonas cedrina* subsp. fulgida subsp. nov., a fluorescent bacterium isolated from the phyllosphere of grasses; emended description of *Pseudomonas cedrina* and description of *Pseudomonas cedrina* subsp. cedrina subsp. nov. Int J Syst Evol Microbiol.

[CR6] Behrens B, Helmer PO, Tiso T, Blank LM, Hayen H (2016). Rhamnolipid biosurfactant analysis using online turbulent flow chromatography–liquid chromatography–tandem mass spectrometry. J Chromatogr A.

[CR7] Berg G, Krechel A, Ditz M, Sikora RA, Ulrich A, Hallmann J (2005). Endophytic and ectophytic potato-associated bacterial communities differ in structure and antagonistic function against plant pathogenic fungi. FEMS Microbiol Ecol.

[CR8] Bible AN, Fletcher SJ, Pelletier DA, Schadt CW, Jawdy SS, Weston DJ, Engle NL, Tschaplinski T, Masyuko R, Polisetti S, Bohn PW, Coutinho TA, Doktycz MJ, Morrell-Falvey JL (2016). A carotenoid-deficient mutant in *Pantoea* sp. YR343, a bacteria isolated from the rhizosphere of *Populus deltoides*, is defective in root colonization. Front Microbiol..

[CR9] Bligh EG, Dyer WJ (1959). A rapid method of total lipid extraction and purification. Can J Biochem Physiol.

[CR10] Borrelli GM, Trono D (2015). Recombinant lipases and phospholipases and their use as biocatalysts for industrial applications. Int J Mol Sci.

[CR11] Brady CL, Cleenwerck I, Venter SN, Engelbeen K, De Vos P, Coutinho TA (2010). Emended description of the genus Pantoea, description of four species from human clinical samples, *Pantoea septica* sp. nov., *Pantoea eucrina* sp. nov., *Pantoea brenneri* sp. nov. and *Pantoea conspicua* sp. nov., and transfer of *Pectobacterium cypripedii* (Hori 1911) Brenner et al. 1973 emend. Hauben et al. 1998 to the genus as *Pantoea cypripedii* comb. nov. Int J Syst Evol Microbiol.

[CR12] Breierova E, Gregor T, Marova I, Certik M, Kogan G (2008). Enhanced antioxidant formula based on a selenium-supplemented carotenoid-producing yeast biomass. Chem Biodivers.

[CR13] Brenes M, Medina E, Romero C, de Castro A (2007). Antimicrobial activity of olive oil. Agro Food Ind Hi Tech.

[CR14] Brown JK (1994). Bootstrap hypothesis tests for evolutionary trees and other dendrograms. Proc Natl Acad Sci USA.

[CR15] Cheng Q (2007). Recent patents on carotenoid production in microbes. Recent Pat Biotechnol.

[CR16] Ciafardini G, Zullo BA (2002). Microbiological activity in stored olive oil. Int J Food Microbiol.

[CR17] Ciafardini G, Zullo BA (2002). Survival of micro-organisms in extra virgin olive oil during storage. Food Microbiol.

[CR18] Ciafardini G, Zullo BA (2018). Virgin olive oil yeasts: a review. Food Microbiol.

[CR19] Ciafardini G, Cioccia G, Zullo BA (2017). Taggiasca extra virgin olive oil colonization by yeasts during the extraction process. Food Microbiol.

[CR20] Criddle CS, DeWitt JT, Grbic-Galic D, McCarty PL (1990). Transformation of carbon tetrachloride by *Pseudomonas* sp. strain KC under denitrification conditions. Appl Environ Microbiol.

[CR21] Cui Y, Fraser C, Gardner G, Huang CJ, Reith M, Windust A (2012). Isolation and optimization of the oleaginous yeast *Sporobolomyces roseus* for biosynthesis of ^13^C isotopically labeled 18-carbon unsaturated fatty acids and *trans* 18:1 and 18:2 derivatives through synthesis. J Ind Microbiol Biotechnol.

[CR22] Dabboussi F, Hamze M, Elomari M, Verhille S, Baida N, Izard D, Leclerc H (1999). Taxonomic study of bacteria isolated from Lebanese spring waters: proposal for *Pseudomonas cedrella* sp. nov. and *P. orientalis* sp. nov. Res Microbiol.

[CR23] Derx HG (1930). Etude sur les Sporobolomycetes. Ann Mycol.

[CR24] Dijk JA, Stams AJM, Schraa G, Ballerstedt H, de Bont JAM, Gerritse J (2003). Anaerobic oxidation of 2-chloroethanol under denitrifying conditions by *Pseudomonas stutzeri* strain JJ. Appl Microbiol Biotechnol.

[CR25] Egamberdieva D, Kucharova Z, Davranov K, Berg G, Makarova N, Azarova T, Chebotar V, Tikhonovich I, Kamilova F, Validov SZ, Lugtenberg B (2011). Bacteria able to control foot and root rot and to promote growth of cucumber in salinated soils. Biol Fert Soils.

[CR26] Eguchi M, Ostrowski M, Fegatella F, Bowman J, Nichols D, Nishino T, Cavicchioli R (2001). *Sphingomonas alaskensis* strain AFO1, an abundant oligotrophic ultramicrobacterium from the North Pacific. Appl Environ Microbiol.

[CR27] El-Agamey A, Lowe GM, McGarvey DJ, Mortensen A, Phillip DM, Truscott TG, Young AJ (2004). Carotenoid radical chemistry and antioxidant/pro-oxidant properties. Arch Biochem Biophys.

[CR28] Erguderet TH, Guven E, Demirer GN (2000). Anaerobic treatment of olive oil waste water in batch reactor. Process Biochem.

[CR29] Ettayebi K, Errachidi F, Jamai L, Tahri-Jouti AM, Sendide K, Ettayebi M (2003). Biodegradation of polyphenols with immobilized *Candida tropicalis* under metabolic induction. FEMS Microbiol Lett.

[CR30] Faraco M, Fico D, Pennetta A, De Benedetto GE (2016). New evidences on efficacy of boronic acid-based derivatization method to identify sugars in plant material by gas chromatography–mass spectrometry. Talanta.

[CR31] Felsenstein J (1981). Evolutionary trees from DNA sequences: a maximum likelihood approach. J Mol Evol.

[CR32] Fiehn O, Kopka J, Trethewey RN, Willmitzer L (2000). Identification of uncommon plant metabolites based on calculation of elemental compositions using gas chromatography and quadrupole mass spectrometry. Anal Chem.

[CR33] Filonow AB (2001). Butyl acetate and yeats interact in adhesion and germination of *Botrytis cinerea* conidia in vitro and in fungal decay of Golden Delicious apple. J Chem Ecol.

[CR34] Fleming HP, Walter WM, Etchells JL (1973). Antimicrobial properties of oleuropein and products of its hydrolysis from green olives. Appl Microbiol.

[CR35] Fraser PD, Pinto MES, Holloway DE, Bramley PM (2000). Application of high-performance liquid chromatography with photodiode array detection to the metabolic profiling of plant isoprenoids. Plant J.

[CR36] Gourama H, Letutour B, Tantaoui-Elaraki A, Benbya M, Bullerman LB (1989). Effects of oleuropein, tyrosol and caffeic acid on the growth of mould isolated from olives. J Food Prot.

[CR37] Gouy M, Guindon S, Gascuel O (2010). SeaView Version 4: a multiplatform graphical user interface for sequence alignment and phylogenetic tree building. Mol Biol Evol.

[CR38] Hou YF, Kong Y, Yang JR, Xin W, Yu HW (2004). Study on immobilization of petroleum biodesulfurization catalyst *Pseudomonas stutzeri* UP-1. Acta Petrol Sin.

[CR39] Jahns T (1995). Purification and properties of urease from *Sporobolomyces roseus*. Antonie Van Leeuwenhoek.

[CR40] Janiyani KL, Wate SR, Joshi SR (1992). Surfactant production by *Pseudomonas stutzeri*. J Microbiol Biotechnol.

[CR41] Joo CS, Oh YS, Chung WJ (2001). Evaluation of bioremediation effectiveness by resolving rate-limiting parameters in diesel-contaminated soil. J Microbiol Biotechnol.

[CR42] Juven B, Henis Y (1970). Studies on the antimicrobial activity of olive phenolic compounds. J Appl Bacteriol.

[CR43] Kim OS, Cho YJ, Lee K, Yoon SH, Kim M, Na H, Park SC, Jeon YS, Lee JH, Yi H, Won S, Chun J (2012). Introducing EzTaxon-e: a prokaryotic 16S rRNA Gene sequence database with phylotypes that represent uncultured species. Int J Syst Evol Microbiol.

[CR44] Kimura M (1980). A simple method for estimating evolutionary rate of base substitutions through comparative studies of nucleotide sequences. J Mol Evol.

[CR45] Kissi M, Mountadar M, Assobhei O, Gargiulo E, Palmieri G, Giardina P (2001). Roles of two white-rot basidiomycete fungi in decolorization and detoxification of olive mill wastewater. Appl Microbiol Biotechnol.

[CR46] Koeuth T, Versalovic J, Lupski JR (1995). Differential subsequence conservation of interspersed repetitive *Streptococcus pneumoniae* BOX elements in diverse bacteria. Genome Res.

[CR47] Koidis A, Triantafillou E, Boskou D (2008). Endogenous microflora in turbid virgin olive oils and the physicochemical characteristics of these oils. Eur J Lipid Sci Technol.

[CR48] Kosikova B, Slavikova E (2004). Biotransformation of lignin polymers derived from beech wood pulping by *Sporobolomyces roseus* isolated from leafy material. Biotechnol Lett.

[CR49] Kumar PSV, Manjunatha BK (2016). Studies on hydrocarbon degradation by the bacterial isolate *Stenotrophomonas rhizophila* (PM-1) from the oil spilled regions of Western Ghats of Karnataka. Sci Technol Arts Res J.

[CR50] Lalucat J, Bennasar A, Bosch R, Garcia-Valdes E, Palleroni NJ (2006). Biology of *Pseudomonas stutzeri*. Microbiol Mol Biol R.

[CR51] Lee DS, Yamada A, Sugimoto H, Matsunaga I, Ogura H, Ichihara K, Adachi S, Park SY, Shiro Y (2003). Substrate recognition and molecular mechanism of fatty acid hydroxylation by cytochrome P450 from *Bacillus subtilis*. Crystallographic, spectroscopic, and mutational studies. J Biol Chem.

[CR52] Lee SO, Kim CS, Cho SM, Choi HJ, Ji GE, Oh DK (2003). Bioconversion of linoleic acid into conjugated linoleic acid during fermentation and by washed cells of *Lactobacillus reuteri*. Biotechnol Lett.

[CR53] Lehmann KB, Neumann RO (1896) Atlas und Grundriss der Bakteriologie und Lehrbuch der speciellen bakteriologischen Diagnostik. München

[CR54] Lo CI, Padhmanabhan R, Mediannikov O, Nguyen TT, Raoult D, Fournier PE, Fenollar F (2015). Genome sequence and description of *Pantoea septica* strain FF5. Stand Genomic Sci.

[CR55] Louws FJ, Fulbright DW, Stephens CT, de Bruijn FJ (1994). Specific genomic fingerprints of phytopathogenic Xanthomonas and Pseudomonas pathovars and strains generated with repetitive sequences and PCR. Appl Environ Microbiol.

[CR56] Maier R (2003). Biosurfactants: evolution and diversity in Bacteria. Adv Appl Microbiol.

[CR57] Margesin R, Labbé D, Schinner F, Greer CW, Whyte LG (2003). Characterization of hydrocarbon-degrading microbial populations in contaminated and pristine Alpine soils. Appl Environ Microbiol.

[CR58] McNicholas S, McDermott H, Power L, Johnson EM, Moroney J, Humphreys H, Smyth EG (2012). *Sporobolomyces roseus* in the cerebrospinal fluid of an immunocompetent patient-to treat or not to treat?. J Med Microbiol.

[CR59] Medina E, Brenes M, Garcia A, Romero C, de Castro A (2009). Bactericidal activity of glutaraldehyde-like compounds from olive products. J Food Prot.

[CR60] Mergaert J, Verdonck L, Kersters K (1993). Transfer of *Erwinia ananas* (synonym, *Erwinia uredovora*) and *Erwinia stewartii* to the Genus *Pantoea* emend. as *Pantoea ananas* (Serrano 1928) comb. nov. and *Pantoea stewartii* (Smith 1898) comb. nov., Respectively, and Description of *Pantoea stewartii* subsp. *indologenes* subsp. nov. Int J Syst Evol Microbiol.

[CR61] Nakase T (2000). Expanding world of ballistosporous yeasts: distribution in the phyllosphere, systematics and phylogeny. J Gen Appl Microbiol.

[CR62] Nelis HJ, De Leenheer AP (1989). Profiling and quantitation of bacterial carotenoids by liquid chromatography and photodiode array detection. Appl Environ Microbiol.

[CR63] Nzila A, Thukair A, Sankara S, Abdur Razzak S (2016). Characterization of aerobic oil and grease-degrading bacteria in wastewater. Environ Technol.

[CR64] Pascazio S, Crecchio C, Ricciuti P, Palese AM, Xiloyannis C, Sofo A (2015). Phyllosphere and carposphere bacterial communities in olive plants subjected to different cultural practices. Int J Plant Biol.

[CR65] Pepi M, Cesàro A, Liut G, Baldi F (2005). An antarctic psychotrophic bacterium *Halomonas* sp. ANT-3b, growing on n-hexadecane, produces a new emulsifying glycolipid. FEMS Microbiol Ecol.

[CR66] Pizzolante G, Cordero C, Tredici SM, Vergara D, Pontieri P, Del Giudice L, Capuzzo A, Rubiolo P, Kanchiswamy CN, Zebelo SA, Bicchi C, Maffei ME, Alifano P (2017). Cultivable gut bacteria provide a pathway for adaptation of *Chrysolina herbacea* to *Mentha aquatica* volatiles. BMC Plant Biol.

[CR67] Pucci OH, Bak MA, Peressutti SR, Klein I, Hartig C, Alvarez HM, Wunsche L (2000). Influence of crude oil contamination on the bacterial community of semiarid soils of Patagonia (Argentina). Acta Biotechnol.

[CR68] Rooney AP, Price NP, Ray KJ, Kuo TM (2009). Isolation and characterization of rhamnolipid-producing bacterial strains from a biodiesel facility. FEMS Microbiol Lett.

[CR69] Sabirova JS, Haddouche R, Van Bogaert I, Mulaa F, Verstraete W, Timmis K, Schmidt-Dannert C, Nicaud J, Soetaert W (2011). The ‘LipoYeasts’ project: using the oleaginous yeast *Yarrowia lipolytica* in combination with specific bacterial genes for the bioconversion of lipids, fats and oils into high-value products. Microb Biotechnol.

[CR70] Saey V, Vanhaeesenbrouck A, Maes S, Van Simaey L, Van Ham L, Deschagt P, Ducatelle R (2011). Granulomatous meningoencephalitis associated with *Sporobolomyces roseus* in a dog. Vet Pathol.

[CR71] Saitou N, Nei M (1987). The neighbour-joining method: a new method for reconstructing phylogenetic trees. Mol Biol Evol.

[CR72] Sambrook J, Russel DW (2001). Molecular cloning: a laboratory manual.

[CR73] Santos DK, Rufino RD, Luna JM, Santos VA, Sarubbo LA (2016). Biosurfactants: multifunctional biomolecules of the 21st century. Int J Mol Sci.

[CR74] Schmidt CS, Alavi M, Cardinale M, Muller H, Berg G (2012). *Stenotrophomonas rhizophila* DSM 14405^T^ promotes plant growth probably by altering fungal communities in the rhizosphere. Biol Fert Soils.

[CR75] Seifert EL, Estey C, Xuan JY, Harper ME (2010). Electron transport chain-dependent mechanisms of mitochondrial H_2_O_2_ emission during long-chain fatty acid oxidation. J Biol Chem.

[CR76] Sijderius R (1946). Heterotrophe bacterien, die thiosulfaat oxydeeren.

[CR77] Singh A, Hamme JD, Ward OP (2007). Surfactants in microbiology and biotechnology: part 2: application aspects. Biotechnol Adv.

[CR78] Smith DD, Nickzad A, Déziel E, Stavrinides A (2016). A novel glycolipid biosurfactant confers grazing resistance upon *Pantoea ananatis* BRT175 against the social amoeba *Dictyostelium discoideum*. mSphere.

[CR79] Sober E (1983). Parsimony in systematics: philosophical issues. Annu Rev Ecol Syst.

[CR80] Suckstorff I, Berg G (2003). Evidence for dose-dependent effects on plant growth by *Stenotrophomonas* strains from different origins. J Appl Microbiol.

[CR81] Talà A, Lenucci MS, Gaballo A, Durante M, Tredici SM, Debowles DA, Pizzolante G, Marcuccio C, Carata E, Piro G, Carpita NC, Mita G, Alifano P (2013). *Sphingomonas cynarae* sp. nov., a proteobacterium that produces an unusual type of sphingan. Int J Syst Evol Microbiol.

[CR82] Van Hamme JD, Singh A, Ward OP (2006). Physiological aspects. Part 1 in a series of papers devoted to surfactants in microbiology and biotechnology. Biotechnol Adv.

[CR83] Vasileva-Tonkova E, Gesheva V (2007). Biosurfactant production by antarctic facultative anaerobe *Pantoea* sp. during growth on hydrocarbons. Curr Microbiol.

[CR84] Verhille S, Batda N, Dabboussi F, Hamze M, Izard D, Leclerc H (1999). *Pseudomonas gessardii* sp. nov. and *Pseudomonas migulae* sp. nov., two new species isolated from natural mineral waters. Int J Syst Bacteriol.

[CR85] Versalovic JSM, De Bruijn FJ, Lupski JR (1994). Genomic fingerprinting of bacteria using repetitive sequence-based polymerase chain reaction. Methods Mol Cell Biol.

[CR86] Vigliotta G, Nutricati E, Carata E, Tredici SM, De Stefano M, Pontieri P, Massardo DR, Prati MV, De Bellis L, Alifano P (2007). *Clonothrix fusca* Roze 1896, a filamentous, sheathed, methanotrophic gammaproteobacterium. Appl Environ Microbiol.

[CR87] Virupakshappa PKS, Krishnaswamy MBM, Mishra G, Mehkri MA (2016). Optimization of crude oil and PAHs degradation by *Stenotrophomonas rhizophila* KX082814 strain through response surface methodology using Box-Behnken design. Biotechnol Res Intern.

[CR88] Walterson AM, Stavrinides J (2015). *Pantoea*: insights into a highly versatile and diverse genus within the Enterobacteriaceae. FEMS Microbiol Rev.

[CR89] White TJ, Bruns T, Lee S, Taylor J, Innis MA, Gelfand DH, Sninsky JJ, White TJ (1990). Amplification and direct sequencing of fungal ribosomal RNA genes for phylogenetics. PCR protocols: a guide to methods and applications. Chapter 38.

[CR90] Wolf A, Fritze A, Hagemann M, Berg G (2002). *Stenotrophomonas rhizophila* sp. nov., a novel plant-associated bacterium with antifungal properties. Int J Syst Evol Microbiol.

[CR91] Yu IS, Kim HJ, Oh DK (2008). Conversion of linoleic acid into 10-hydroxy-12(*Z*)-octadecenoic acid by whole cells of *Stenotrophomonas nitritireducens*. Biotechnol Prog.

[CR92] Zullo BA, Ciafardini G (2008). Lipolytic yeasts distribution in commercial extra virgin olive oil. Food Microbiol.

[CR93] Zullo BA, Cioccia G, Ciafardini G (2010). Distribution of dimorphic yeast species in commercial extra virgin olive oil. Food Microbiol.

