# Supplementary material for: Characterization of two Pantoea strains isolated from extra-virgin olive oil
Source: AMB Express. 2018 Jul 10;8:113. doi: 10.1186/s13568-018-0642-z (PMC6039349; doi:10.1186/s13568-018-0642-z)
Supplement: Supplementary file 1 — Additional file 1. Additional tables and figures. [file 13568_2018_642_MOESM1_ESM.pdf]

Additional Material

AMB Express

Characterization of two *Pantoea* strains isolated from extra-virgin olive oil

Graziano Pizzolante[1], Miriana Durante[2], Daniela Rizzo[3], Marco Di Salvo[1], Salvatore Maurizio Tredici[1], Maria Tufariello[2], Angelo De Paolis[2], Adelfia Talà[1], Giovanni Mita[2], Pietro Alifano[1#], Giuseppe Egidio De Benedetto[3#]

[1]Department of Biological and Environmental Sciences and Technologies (DiSTeBA), University of Salento, Via Provinciale Monteroni 165, 73100 - Lecce, Italy; [2]Istituto di Scienze delle Produzioni Alimentari - CNR, Via Provinciale Monteroni 165, 73100 - Lecce, Italy; [3]Laboratory of Analytical and Isotopic Mass Spectrometry, Department of Cultural Heritage, University of Salento, Lecce, Italy

#To whom correspondence should be addressed:

Pietro Alifano, Tel: 0039 0832 298856; e-mail: pietro.alifano@unisalento.it

Giuseppe E. De Benedetto, Tel: 0039 0832 299067; e-mail: giuseppe.debenedetto@unisalento.it

Table S1. Phenotypic traits of *Pantoea septica* OOWS-10, OOYS-10 and related type strain.

Strains: 1, *Pantoea septica* OOWS-10 ; 2, *Pantoea septica* OOYS-10 ; 3, *Pantoea septica* LMG 5345^T (Brady et al. 2010). +, positive reaction; -, negative reaction, (+); weakly positive reaction; ND, not determined. W, white; Y, yellow; B, beige.[a]

**Tests**	**1**	**2**	**3**
Colony colour	W	Y	B
Motility	+	+	+
**Enzymatic activities:**			
Oxidase	-	-	-
Catalase	-	-	-
Arginine dehydrolase	-	-	-
Lysine decarboxylase	-	-	-
Ornithine decarboxylase	-	-	-
Phenylalanine deaminase	(+)	(+)	(+)
**Production of:**			
Indole	-	-	-
H_2S	-	-	-
Acetoin	+	+	+
Nitrite	+	+	ND
**Hydrolysis of:**			
Gelatin	-	-	ND
Urea	-	-	-
**Acid from:**			
Amygdalin	+	+	ND
L-Arabinose	+	+	+
Dulcitol	-	-	-
D-Glucose	+	+	ND
Inositol	+	+	ND
Lactose	+	+	+
D-Mannitol	+	+	+
Melibiose	+	+	ND
L-Rhamnose	+	+	+
Sorbitol	+	+	ND
Saccharose	+	+	ND
**Assimilation of:**			
L-Arabinose	+	+	+
Capric acid	-	-	-
Citric acid	-	-	+
Gluconate	+	+	+
D-Glucose	+	+	+
D-Malate	+	+	+
D-Maltose	+	+	+
D-Mannitol	+	+	+
D-Mannose	+	+	+
N-acetyl-Glucosamine	+	+	+

[a]Data source:

Brady CL, Cleenwerck I, Venter SN, Engelbeen K, De Vos P & Coutinho TA (2010) Int J Syst Evol Microbiol 60:2430-2440.

Table S2 Phenotypic traits of *Stenotrophomonas rhizophila* OOOWS-2 and OOOWS-9 and related reference strain. The two isolated strains were not able to utilize (as carbon source): adipic acid, D- and L-arabinose, capric acid, dulcitol, erythritol, D-and L-fucose, glycerol, inulin, D-melezitose, methyl-αD-glucopyranoside, methyl-αD-mannopyranoside, methyl-βD-xylopyranoside, phenylacetic acid, potassium gluconate, potassium 2-ketogluconate, potassium 5-ketogluconate, D-raffinose, L-rhamnose, D-ribose, D-sorbitol, L-sorbose, starch, D-tagatose. Strains: 1, *Stenotrophomonas rhizophila* OOOWS-2; 2, *Stenotrophomonas rhizophila* OOOWS-9; 3, *Stenotrophomonas rhizophila* DSM 14405^{T} (Wolf et al. 2002). +, positive reaction; -, negative reaction, (+); weakly positive reaction; ND, not determined. OW, opaque white; Y, yellow.[a]

**Tests**	**1**	**2**	**3**
Colony colour	OW	OW	Y
Motility	+	+	+
**Enzymatic activities:**			
Oxidase	-	-	-
β-Galactosidase	+	+	ND
Arginine dehydrolase	-	-	-
**Production of:**			
Nitrite	(+)	(+)	+
**Hydrolysis of:**			
Aesculin	+	+	+
Gelatin	+	+	+
Urea	-	-	-
**Acid from:**			
D-Glucose	-	-	-
**Assimilation of:**			
N-acetyl-Glucosamine	+	+	+
D-Adonitol	+	+	ND
Amygdalin	+	+	ND
D-Arabitol	+	+	ND
L-Arabitol	+	+	ND
Arbutin	+	+	ND
D-Cellobiose	+	+	+
Citric acid	+	+	+
D-Fructose	+	+	+
D-Galactose	+	+	+
Gentioniose	+	+	+
D-Glucose	+	+	+
Glycogen	+	+	+
Inositol	+	+	ND
D-Lactose	+	+	+
D-Lyxose	+	+	ND
D-Malate	+	+	+
D-Maltose	+	+	+
D-Mannitol	+	+	ND
D-Mannose	+	+	+
D-Melibiose	+	+	ND
D-Saccharose	+	+	+
Salicin	+	+	+
D-Trehalose	+	+	+
D-Turanose	+	+	+
Xylitol	+	+	ND
D-Xylose	+	+	+
L-Xylose	+	+	+

[a]Data source:

Wolf A, Fritze A, Hagemann M, Berg G (2002) Int J Syst Evol Microbiol 52:1937-1944.

Table S3 Phenotypic traits of *Pseudomonas* sp. OOBS-2 and related reference strains. The isolated strain was not able to utilize (as carbon source): adipic acid, amygdalin, D-arabinose, arbutin, D-cellobiose, dulcitol, D- and L-fucose, gentiobiose, glycogen, inulin, D-lactose, D-maltose, D-melezitose, methyl-αD-glucopyranoside, methyl-αD-mannopyranoside, methyl-β-D-xylopyranoside, phenylacetic acid, D-raffinose, L-rhamnose, salicin, D-sorbitol, L-sorbose, starch, D-tagatose, L-xylose.

Strains: 1, *Pseudomonas* sp. OOBS-2; 2, *Pseudomonas gessardii* CIP 105469^T (Verhille et al. 1999); 3, *Pseudomonas cedrina* subsp. *cedrina* CIP 105541^T (Dabboussi et al. 1999; Behrendt et al. 2009). +, positive reaction; -, negative reaction, (+); weakly positive reaction; ND, not determined. B, brown; NP, non pigmented.[a]

**Tests**	**1**	**2**	**3**
Colony colour	B	ND	NP
Motility	+	+	+
**Enzymatic activities:**			
Oxidase	+	+	+
β-Galactosidase	-	-	-
Arginine dehydrolase	+	-	+
**Production of:**			
Nitrite	(+)	+	+
**Hydrolysis of:**			
Aesculin	-	-	-
Gelatin	+	-	+
Urea	(+)	-	-
**Assimilation of:**			
D-Adonitol	+	+	+
N-acetyl-Glucosamine	+	+	+
D-Arabitol	+	+	+
L-Arabitol	+	+	ND
Capric acid	+	+	+
Citric acid	+	+	+
D-Fructose	+	+	+
D-Galactose	+	+	+
D-Glucose	+	+	+
Inositol	+	+	+
D-Lyxose	+	-	+
D-Malate	+	+	+
D-Mannose	+	+	+
D-Mannitol	+	+	+
D-Melibiose	+	-	-
Phenylacetic acid	+	-	-
Potassium gluconate	+	+	+
Potassium 2-ketogluconate	+	ND	+
Potassium 5-ketogluconate	+	ND	-
D-Saccharose	+	-	-
D-Trehalose	+	+	+
D-Turanose	+	-	-
Xylitol	+	+	ND

[a]Data source:

Behrendt U, Schumann P, Meyer JM, Ulrich A (2009) Int J Syst Evol Microbiol 59:1331-1335.

Dabboussi F, Hamze M, Elomari M, Verhille S, Baida N, Izard D, Leclerc H (1999) Res Microbiol 150:303-316.

Verhille S, Batda N, Dabboussi F, Hamze M, Izard D, Leclerc H (1999) Int J Syst Bacteriol 49:1559-1572.

Table S4 Phenotypic traits of *Pseudomonas stutzeri* OOYW-9 and related type strain. The isolated strain was not able to utilize (as carbon source): N-acetyl-glucosamine, D-adonitol, amygdalin, D- and L-arabinose, L-arabitol, arbutin, D-cellobiose, dulcitol, erythritol, D- and L-fucose, D-galactose, gentiobiose, inositol, inulin, D-lactose, D-lyxose, D-mannose, D-melezitose, D-melibiose, methyl-αD-glucopyranoside, methyl-αD-mannopyranoside, methyl-βD-xylopyranoside, phenylacetic acid, potassium 2-ketogluconate, D-raffinose, L-rhamnose, D-ribose, D-saccharose, salicin, D-sorbitol, L-sorbose, D-tagatose, D-trehalose, D-turanose, xylitol.

Strains: 1, *Pseudomonas stutzeri* OOYW-9; 2, *Pseudomonas stutzeri* ATCC 17588[T] (Lehmann and Neumann 1896; Sijderius 1946); 3, *Pseudomonas balearica* DSM 6083[T] (Bennasar et al. 1996). +, positive reaction; -, negative reaction; (+), weakly positive reaction, d, diverse reaction; ND, not determined. Y, yellow; P, pale; NP, non pigmented.[a]

**Tests**	**1**	**2**	**3**
Colony colour	Y	P	NP
Motility	+	+	+
**Enzymatic activities:**			
Oxidase	+	+	+
β-Galactosidase	-	-	-
Arginine dehydrolase	-	-	-
**Production of:**			
Nitrite	+	+	+
**Hydrolysis of:**			
Aesculin	-	-	-
Gelatin	-	-	-
Urea	-	-	-
**Acid from:**			
D-Glucose	-	-	-
**Assimilation of:**			
D-Arabitol	+	ND	ND
Capric acid	+	d	ND
Citric acid	+	+	ND
D-Fructose	+	d	ND
D-Glucose	+	+	ND
Glycerol	+	+	ND
Glycogen	+	-	ND
D-Malate	+	+	+
D-Maltose	+	+	+
D-Mannitol	+	d	-
Potassium gluconate	+	d	ND
Starch	+	+	+
D-Xylose	-	-	+
L-Xylose	-	-	+

[a]Data source:

Bennasar A, Rossellò-Mora R, Lalucat J, Moore ERB (1996) Int J Syst Evol Microbiol 46:200-205.

Lehmann KB, Neumann RO (1896) Atlas und Grundriss der Bakteriologie und Lehrbuch der speciellen bakteriologischen Diagnostik. München.

Sijderius R (1946) Heterotrophe bacterien, die thiosulfaat oxydeeren. Thesis. Amsterdam: University Amsterdam.

Table S5 Phenotypic traits of *Sporobolomyces roseus* OOPS-1, OOPS-10 and related reference strain. Strains: 1, *Sporobolomyces roseus* OOPS-1; 2, *Sporobolomyces roseus* OOPS-10; 3, *Sporobolomyces roseus* MUCL 30251^T (Kluyver 1925). +, positive reaction; -, negative reaction, (+), weakly positive reaction; ND, not determined. P, pink.[a]

**Tests**	**1**	**2**	**3**
Colony colour	P	P	P
**Assimilation of:**			
D-Adonitol	-	-	-
L-Arabinose	-	-	-
Calcium 2-keto gluconate	-	-	-
D-Cellobiose	+	+	+
D-Galactose	+	+	+
D-Glucose	+	+	+
Glycerol	+	+	+
Inositol	-	-	-
D-Lactose	-	-	-
D-Maltose	+	+	+
D-Melezitose	+	+	+
Methyl-αD-glucopyranoside	(+)	(+)	d
N-acetyl-glucosamine	-	-	-
D-Raffinose	+	+	+
D-Saccharose	+	+	+
D-Sorbitol	(+)	(+)	ND
D-Trehalose	+	+	+
D-Xylitol	-	-	-
D-Xylose	(+)	(+)	d

[a]Data source:

Kluyver AJ (1925) Uber Spiegelbilder erzeugende Hefenarten und die Hefengattung *Sporobolomyces*. Zentralbl Bakteriol, II Abt 63:1-20.

Table S6. Growth of microbial isolates with Alài® extra-virgin olive oil as sole carbon and energy source.

Microbial isolate	Growth on solid M9-OO	Growth on solid M9-glucose	μ (h^{-1}) in liquid M9-OO[a]	μ (h^{-1}) in liquid M9-glucose[a]
Pantoea septica OOWS-10	+++	++++	0.25	0.48
Pantoea septica OOYS-10	+++	++++	0.30	0.50
Stenotrophomonas rhizophila OOOWS-2	++	++++	0.15	0.53
Stenotrophomonas rhizophila OOOWS-9	++	++++	0.18	0.56
Pseudomonas stutzeri OOYW-9	+++	++++	0.20	0.45
Pseudomonas cedrina OOBS-2	+	++++	0.10	0.47
Sporobolomyces roseus OOPS-1	+++	++++	0.40	0.51
Sporobolomyces roseus OOPS-10	+++	++++	0.37	0.49

[a] μ, specific growth rate during the logarithmic phase. Values are means of five independent experiments. **+,** very poor growth; ++, poor growth; +++, good growth; ++++, very good growth.

Table S7. Fatty acid profiles from *Pantoea septica* isolates OOYS-10 and OOWS-10[a].

**Fatty acid**	***Pantoea septica* OOWS-10**	***Pantoea septica* OOYS-10**
	% of total fatty acids	
$C_{14:0}$	3.8	10.1
$C_{15:0}$	1.3	6.7
$C_{16:0}$	34.7	32.9
$C_{18:0}$	2.3	3.3
$C_{17:0\ cyclo}$	8.8	4.3
$C_{16:1\omega7c}$	26.5	22.3
$C_{18:1\omega7c}$	22.6	20.4

[a]Values are percentage of total fatty acids. Components representing less than 0.5% in all strains were omitted.

Table S8. Carotenoids and isoprenoid quinones from *Pantoea septica* isolates OOYS-10 and OOWS-10[a]

	***Pantoea septica* OOWS-10**	***Pantoea septica* OOYS-10**
	$\mu g\ g^{-1}$ dw	
**Carotenoids**		
Lutein	0.8±0.02	1.6±0.2
ß carotene	0.7±0.01	1.2±0.1
**Isoprenoid quinones**		
Quinone 8 (Q-8)	109.9±1.1	199.9±1.8
Quinone 10 (Q-10)	2.3±0.1	6.3±0.7

[a]Values are expressed as $\mu g\ g^{-1}$ dw.

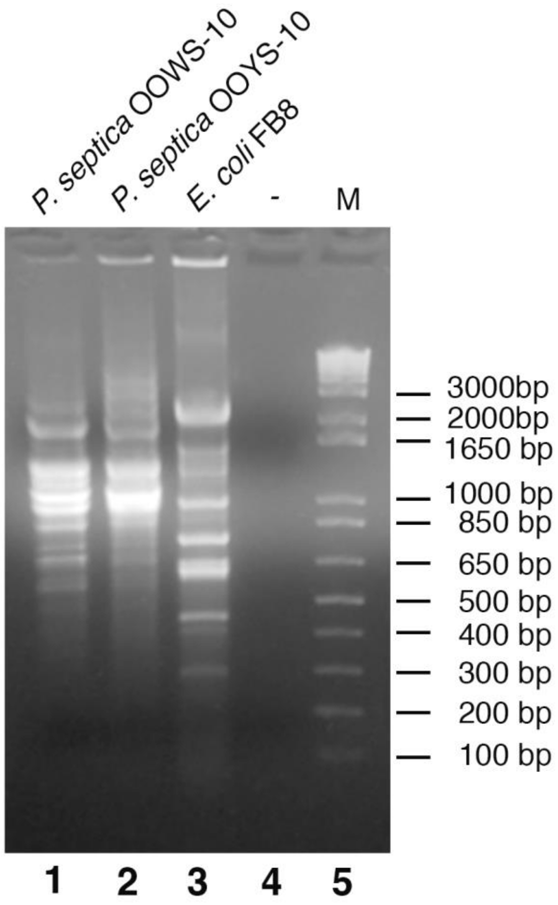

Fig. S1. BOX-PCR genomic fingerprinting of *P. septica* isolates OOWS-10 and OOYS-10. Bacterial DNA was extracted from *P. septica* isolates OOWS-10 (lane 1) and OOYS-10 (lane 2) and reference *E. coli* K12 strain FB8 (lane 3), and BOX-PCR genomic fingerprinting was carried out as described in the Material and Methods section. Negative control (-, no DNA) and molecular weight ladders (M) were loaded, respectively, in lanes 4 and 5.

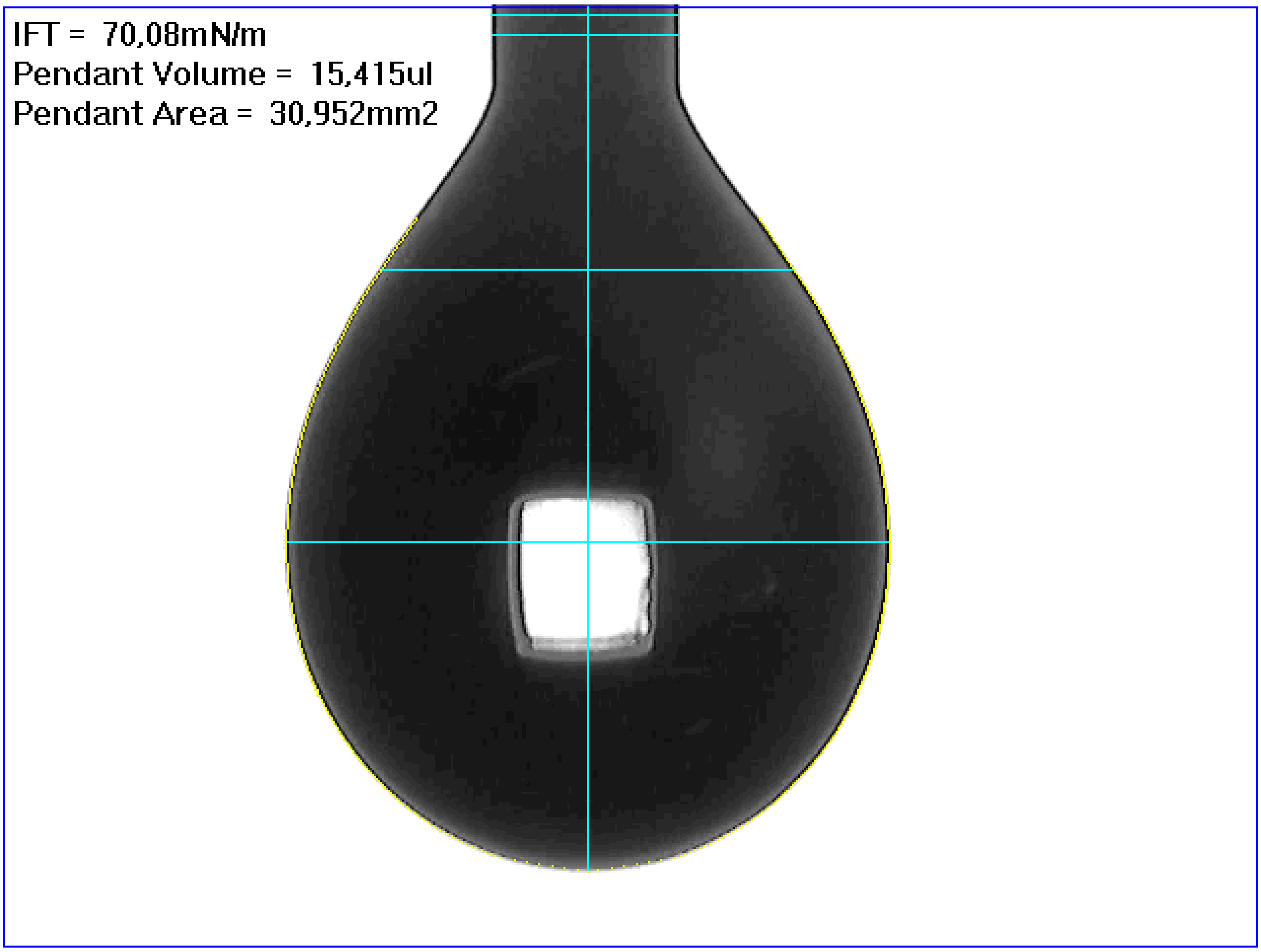

Fig. S2. Surface tension measurements with pendant drop tensiometry: grayscale image, drop data and resulting surface tension for the PBS control.

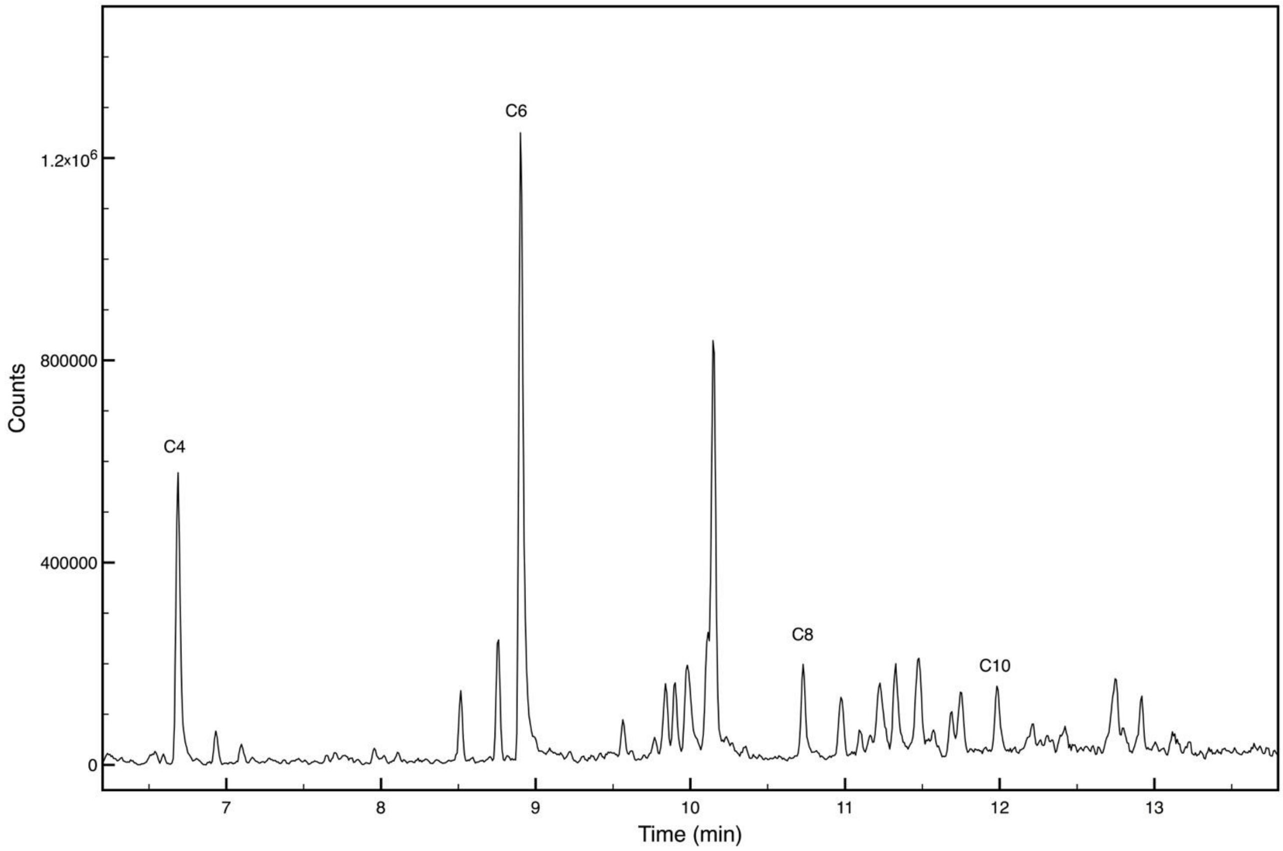

Fig. S3. Extracted ion chromatogram in the 250-350 mass range where the identified ß-hydroxy fatty acids have been labelled.
